# Emergent Self‐Adaptation in an Integrated Photonic Neural Network for Backpropagation‐Free Learning

**DOI:** 10.1002/advs.202404920

**Published:** 2024-11-20

**Authors:** Alessio Lugnan, Samarth Aggarwal, Frank Brückerhoff‐Plückelmann, C. David Wright, Wolfram H. P. Pernice, Harish Bhaskaran, Peter Bienstman

**Affiliations:** ^1^ Photonics Research Group Ghent University‐imec Ghent 9052 Belgium; ^2^ Department of Materials University of Oxford Parks Road Oxford OX1 3PH UK; ^3^ Department of Physics CeNTech, University of Münster Heisenbergstraße 48149 Münster Germany; ^4^ Department of Engineering University of Exeter Exeter EX4 4QF UK

**Keywords:** neuromorphic computing, machine learning, phase change materials, reservoir computing, self‐adapting systems, silicon photonics, synaptic plasticity

## Abstract

Plastic self‐adaptation, nonlinear recurrent dynamics and multi‐scale memory are desired features in hardware implementations of neural networks, because they enable them to learn, adapt, and process information similarly to the way biological brains do. In this work, these properties occurring in arrays of photonic neurons are experimentally demonstrated. Importantly, this is realized autonomously in an emergent fashion, without the need for an external controller setting weights and without explicit feedback of a global reward signal. Using a hierarchy of such arrays coupled to a backpropagation‐free training algorithm based on simple logistic regression, a performance of 98.2% is achieved on the MNIST task, a popular benchmark task looking at classification of written digits. The plastic nodes consist of silicon photonics microring resonators covered by a patch of phase‐change material that implements nonvolatile memory. The system is compact, robust, and straightforward to scale up through the use of multiple wavelengths. Moreover, it constitutes a unique platform to test and efficiently implement biologically plausible learning schemes at a high processing speed.

## Graphical abstract

A novel integrated photonic neural network with self‐adaptive behavior is demonstrated, which interfaces the space, time and frequency domains. The network nodes feature both multi‐scale volatile memory and non‐volatile memory, exploiting phase change materials. The network parameters can be modified by non‐volatile adaptation to the input, similarly as in the brain, which can be leveraged for scalable biologically‐plausible learning.

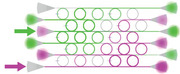

## 1 Introduction

In recent years, computational power and applicability of artificial neural networks (ANNs) have grown rapidly, to the point that this technology is taking a more and more important role in many different fields and aspects of society.^[^ ^1^, ^2^ ^]^ However, the mainstream approach of simulating ANNs in software is highly inefficient because of the large number of parallel operations required for inference and training.^[^ ^3^, ^4^, ^5^ ^]^ On the other hand, biological brains show us that more versatile, more powerful and continuously learning neural networks exist that are extremely energy efficient. Still, there are many unknowns regarding the mechanisms of learning and memorizing in our brain, and today's ANN models are based on an extremely simplified abstraction of the brain's behavior.^[^ ^6^ ^]^

An example of a research path striving to correct this mismatch is the search for biologically plausible learning rules.^[^ ^6^, ^7^, ^8^, ^9^, ^10^, ^11^ ^]^ This search mainly originates from the evidence that backpropagation (BP), the pillar of conventional training approaches, is not likely to happen in biological neural networks. Therefore, researchers in the field are looking for biologically plausible learning mechanisms to obtain powerful and efficient ANNs. In particular, plastic self‐adaptation is a central property in this regard, as it is considered to be the main enabling mechanism behind memory and learning in biological brains.^[^ ^12^ ^]^ We consider a network “plastic” when the response of its components (nodes, connections) to their input depends on the history of this input, in a non‐volatile way w.r.t. the relevant timescales. Importantly, thanks to plastic self‐adaptation, a suitably designed physical neural network can learn to perform useful functions just by plastically adapting to its inputs in an autonomous way, without the need for an external controller setting the plastic weights. Although there are still no powerful training algorithms nor ANN architectures able to fully achieve this brain‐like type of learning on hardware, we agree that plasticity‐based learning and self‐adaptation is very likely to play a fundamental role in future development of large‐scale neuromorphic hardware. In fact, intense research effort is being spent both to achieve it in ANNs^[^ ^13^ ^]^ and, in parallel, to better understand related mechanisms in biological neural networks.^[^ ^6^, ^9^ ^]^

An attractive feature of such self‐adaptation is that it could alleviate a major scalability issue in hardware implementations of ANNs. Indeed, current state‐of‐the‐art training approaches require full and precise tuning of network parameters and observability of internal states (as it is demanded by BP and gradient descent). In hardware implementations this implies access to the internal components of an ANN through physical connections and control devices, making it extremely hard to actually scale these systems up to numbers of synapses and neurons comparable to the ones in biological brains. However, learning by plastic self‐adaptation would remove this limitation, since the network connections would be used at the same time both to process input information and to train the network parameters.

In this work we provide a key step forward in development of autonomous self‐adapting neuromorphic computing, by experimentally realizing for the first time a scalable hardware ANN whose physical nodes exhibit both volatile memory (short‐term plasticity) and non‐volatile memory (long‐term plasticity), providing at the same time high computing power and the possibility of efficient training through self‐adaptation. Importantly, the network's plastic behavior is fully emergent in the sense that it does not rely on an external controller updating the synaptic weights, or on a global reward signal that is explicitly fed back into the network. Our system is implemented in silicon photonics,^[^ ^14^ ^]^ a compact and industry compatible technology to create chip‐based optical networks. In order to realize the plasticity, we use phase change materials (PCMs), whose properties can be modified in a nonvolatile way using optical pulses.

Compared with electronics‐based or other neuromorphic computing platforms,^[^ ^15^, ^16^, ^17^ ^]^ photonics offers unique advantages in terms of parallelism, energy efficiency, latency and bandwidth of interconnects.^[^ ^13^, ^18^, ^19^ ^]^ These are particularly relevant for the development of large‐scale hardware ANNs, which comprise a huge number of parallel weighted connections (synapses). Such advantages ultimately arise from the intrinsic difference in the physics behind signal propagation: differently from current‐based signals conveyed by an electronic connection, photons traveling through a dielectric medium do not directly interact with each other. This enables the transmission of multiple signals in parallel through the same channel by using light of different wavelengths (i.e., WDM, short for “wavelength division multiplexing”). This can happen at high speeds and with low energy loss. On the other hand, for the very same reason, nonlinearity and memory have been notoriously difficult to implement efficiently in photonics.

Recently, however, phase change materials (PCMs) have been shown to introduce all optical non‐volatile memory, and thus physical plasticity, into integrated photonics with relatively high energy efficiency and speed.^[^ ^20^, ^21^ ^]^ In particular, chalcogenide alloys such as GST (short for Ge_2_Sb_2_Te_5_) can be deposited in thin films on top of integrated photonic waveguides, whose optical absorption and refractive index depend significantly on the PCM memory state, which is in turn determined by how much of the PCM is in the amorphous state or in the crystalline state. Specifically, infrared light absorption by crystalline GST is much higher compared to amorphous GST. Importantly, powerful enough optical pulses traveling through the waveguide can quickly heat and melt the PCM layer, whose final non‐volatile state will depend on how fast the optical heating decays: slow cooling allows the melted PCM to crystallize, while fast cooling leaves it in the amorphous state. Typical optical pulses used for memory switching have peak powers of a few tens of milliwatts and durations of tens to hundreds of nanoseconds. In this work, we employ GST layers to introduce all‐optical cascadable memory, and thus long‐term plasticity, in an integrated photonic ANN.

Although photonics and PCMs have been used to build neuromorphic systems before, they mainly rely on an external control scheme that explicitly sets the weights. As such, they can be described only as plastic in the very narrow sense that they can be changed, but they lack autonomous emergent behavior. Moreover, current state‐of‐the‐art approaches still have some additional drawbacks. Indeed, the difficulty of fabricating efficient and cascadable nonlinear nodes is still a major impediment to the scalability of neuromorphic photonics systems.^[^ ^18^ ^]^ This challenge has been tackled, for instance, by employing all‐optical PCM switching in order to obtain a threshold‐like nonlinearity on optical input pulses at different wavelengths.^[^ ^21^ ^]^ However, this approach requires separate optical pulse generation for the input and output of a neuron, and a dedicated operation cycle to reset the PCM state after a neuron activation, making the employment of many cascaded neurons challenging in practice. In contrast, in this article we present a fully autonomous recurrent neural network capable of processing sequential data, whose nodes concurrently provide nonlinearity, multi‐scale volatile memory and plastic self‐adaptation. Another popular approach to build artificial neurons is exploiting the nonlinearity arising from converting optical signals into electric ones by means of a photodetector.^[^ ^22^, ^23^, ^24^ ^]^ In order to cascade multiple neurons of this type, the signal can be reconverted to the optical domain by means of a modulator. Nevertheless, this approach presents evident scalability issues, such as a relatively large neuron footprint, high complexity and copious metal wiring. Moreover, similarly to the aforementioned approach based on PCM, every neuron layer requires two dedicated optical input channels. Furthermore, an important general challenge is to cascade multiple neuron layers in a photonic integrated ANN, which can be trained in situ and online.^[^ ^25^ ^]^ Indeed, state‐of‐the‐art efforts toward this direction managed to deploy only a quite limited number of neurons and layers.^[^ ^26^, ^27^ ^]^ Again, as mentioned before, in these artificial photonic neurons, autonomously emerging plasticity is hardly ever considered, especially in the context of scalable networks. Even outside the field of photonics, e.g., considering the more mature electronics‐based neuromorphic hardware, most experimental works about self‐adaptive neuromorphic computing are still about single components (such as an artificial neuron or a synapse) rather than full ANNs.^[^ ^13^ ^]^ Still, self‐adaptation is considered to be an essential challenge and opportunity for future research.

In this work, we present an experimental realization of plastic photonic neurons in scalable arrays. We combine for the first time the volatile nonlinear dynamics of silicon microring resonators (MRRs) and the non‐volatile memory provided by PCM cells, in order to create an autonomously self‐adapting dynamical system. In addition, we pair this with a novel cascaded architecture based on simple linear regression, where the most promising plastic adaptations are selected and combined. The training is backpropagation‐free and vastly simplified compared to, e.g., backpropagation in deep neural networks. Moreover, the system naturally lends itself to WDM exploitation, such that the cascading does not come at the expense of on‐chip footprint. Importantly, the proposed neuromorphic hardware can be trained and used as a testing platform for biologically compatible training procedures based on plastic self‐adaptation and recurrent dynamics, as we discuss later on. As a test for its learning and inference capabilities, we show that the system, combined with a novel backpropagation‐free training scheme, achieves a particularly good accuracy of 98.2% on the MNIST^[^ ^28^ ^]^ task, a popular benchmark task looking at classification of written digits.

In Section 2, we introduce the proposed type of integrated photonic network and its main properties. In Section 3, we then present an investigation on the emergent network plasticity properties for a highly nonlinear time series classification task, using purposely constructed pulse sequences to trigger plasticity. In Section 3, building on this knowledge, we subsequently introduce a hierarchical network architecture continuously operating in the plastic regime without requiring specially constructed training sequences. This is coupled to a simple BP‐free learning scheme that amplifies the most promising autonomously emerging plastic adaptations. As an example application, we show that the network response can be used to achieve high accuracy of 98.2% on the popular MNIST benchmark task. In the *Discussion* section, we explore the scalability of the proposed hardware neuromorphic platform and the relation to existent biologically plausible algorithms, like FF (Forward–Forward) and DFA (Direct Feedback Alignment). The Supporting Information contains further material regarding the single building block (MRR), the investigation of the plasticity property and our machine learning approach.

## 2 A Scalable Photonic Recurrent Neural Network with Emergent Synaptic Plasticity

**Figure 1:**
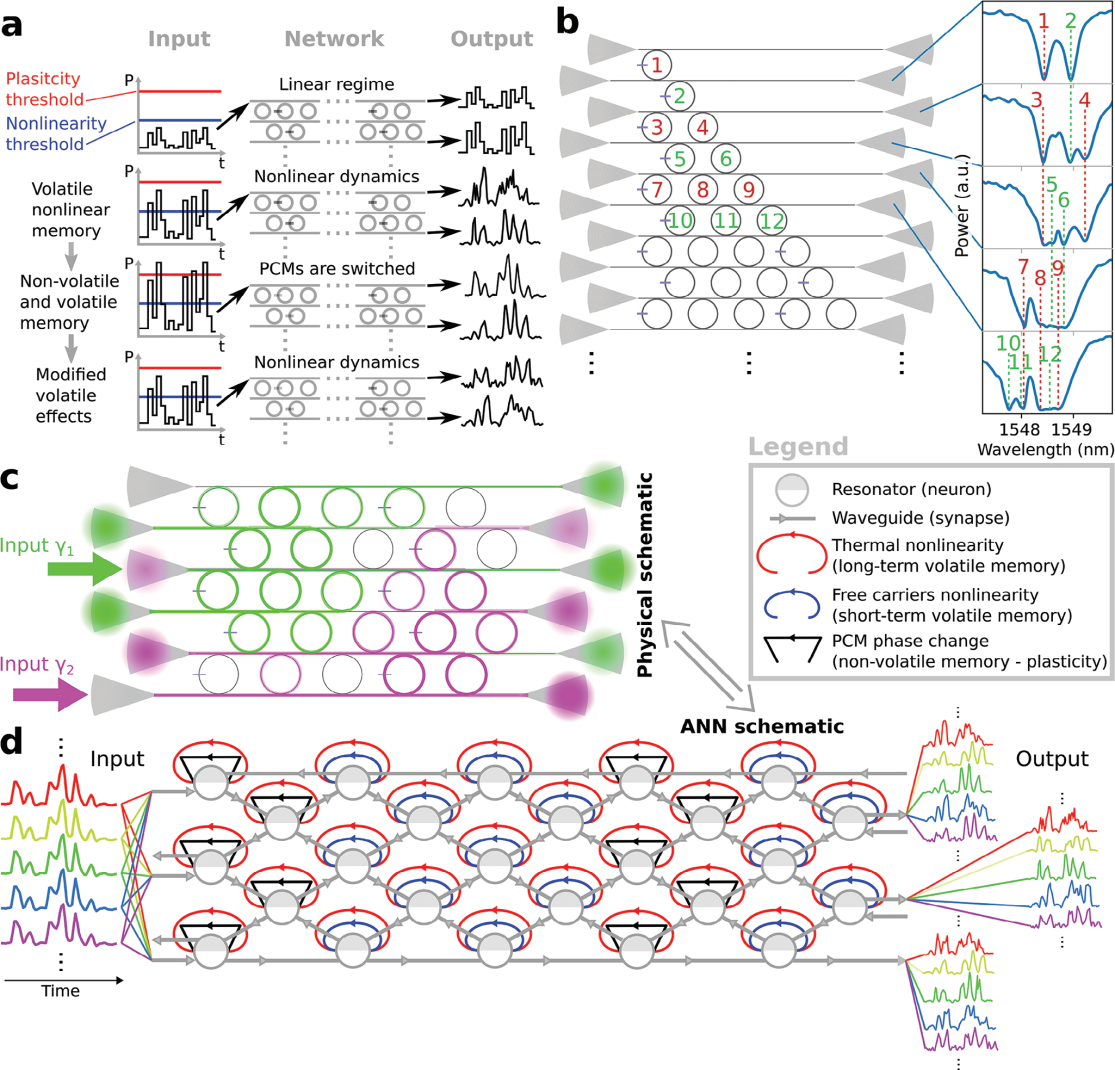
Photonic plastic recurrent resonator neural network (PPRRNN). a) Basic functionality of a PPRRNN. *First row*: a low‐power input waveform does not trigger nonlinear dynamics, thus resulting in a linear network response (no distortion on the output waveforms). *Second row*: a powerful enough input waveform can excite nonlinear dynamics in the PPRRNN, so that different nonlinear representations of the input are obtained at different output ports (and at different wavelengths). *Third row*: a further increase in input power can trigger non‐volatile changes in the PPRRNN, due to PCM switching. *Forth row*: setting the same input power as in the second row results in different output nonlinear representations because of the previous non‐volatile changes (see third row). These properties enable network training through plastic self‐adaptation. b) Design of a triangular PPRRNN and measured optical spectra at the indicated output ports, each corresponding to a low‐power input inserted through the grating coupler to the left on the same straight waveguide. The numbers indicate which nodes correspond to which resonant dips in the spectra. c) Possible light distribution in two “virtual” networks occurring in a rectangular PPRRNN by exciting it with coherent light at two different wavelengths and at two different input ports. d) Corresponding equivalent ANN schematic showing optical connections (grey) and recurrent connections associated with different nonlinear effects in the network nodes excited by light propagation (see the legend).

We present a compact and simple (in terms of design and fabrication) integrated photonic circuit that mimics several key properties of biological neural networks. We now explain its main operational characteristics, arising from a balance between volatile and non‐volatile all‐optical nonlinear memory. The system takes as input and returns as output multiple time‐dependent optical signals. If the input power is high enough (over the *nonlinearity threshold* but below the *plasticity threshold*, see **Figure** **1**a), the corresponding outputs consist of nonlinear transformations with memory (here also referred to as *representations*) of the input, resulting from complex multiphysics dynamics occurring in our photonic network. Such a network activity does not modify the behavior of the nodes in a persistent way. However, increasing the power above the plasticity threshold, results in nonvolatile changes of the network response, that persist when the power is decreased again below the plasticity threshold. Important to realize is that the exact plastic changes depend on the time evolution of the light intensities inside the different nodes. These in turn depend in a nontrivial way on the input sequence sent into the system, which is subject to all the nonlinear resonances inside the MRRs. As such, we have created a system that can autonomously modify its behavior in an emergent fashion based on the inputs it receives, and is able to encode this in long‐term memory. This way, as we will demonstrate in Section 3, multiple and diverse permanent network modifications can be obtained by means of different input signals.

Additionally, our photonic neural network can take in several time‐dependent inputs with different optical wavelengths at the same time (WDM), at each physical port, while producing as many output signals at each output port. This greatly increases the network computational power and throughput. Conveniently, in the same circuit and depending on the employed wavelength, different wavelengths can either be coupled together by the photonic neurons, so as to expand the effective network dimension, or they can be processed separately and concurrently by different subnetworks (consisting of disjoint sets of neurons) forming spontaneously, so as to carry out multiple tasks at the same time. In the rest of this section we will go into more technical details in order to explain how the described network properties arise.

The building blocks of the neuromorphic hardware are simple, compact and mature photonic devices, namely silicon microring resonators (MRRs),^[^ ^29^ ^]^ which we drive into a nonlinear regime in order to achieve cascadable nonlinear nodes with multi‐scale volatile memory.^[^ ^30^, ^31^, ^32^ ^]^ To do so, we exploit the competing effect of variations in temperature and free carrier concentration (with timescales of respectively a few hundred nanoseconds and a few nanoseconds) triggered by optical input signals. At the same time, for the non‐volatile memory, we benefit from the power concentration and the enhanced sensitivity to perturbations granted by the resonant behavior of MRRs. This allows us to achieve a sufficient optical contrast with relatively short PCM patches, thus obtaining more efficient and faster memory operations.^[^ ^33^ ^]^ That way, we can introduce cascadable and efficient all‐optical non‐volatile memory nodes into our photonic ANN. In the past, MRRs have been successfully employed to build synapses or neurons in neuromorphic computing applications,^[^ ^34^ ^]^ as well as integrated photonic PCM devices.^[^ ^21^, ^35^, ^36^ ^]^ In this work, we combine for the first time nonlinear dynamics of silicon MRRs and non‐volatile memory provided by PCM cells, to build a hardware ANN with multiscale volatile memory and emergent plasticity.

The network architecture we propose, which we call *photonic plastic recurrent resonator neural network* (PPRRNN), is composed of an arrangement of nonlinear nodes (bare silicon MRRs) and plastic nodes (silicon MRRs with PCM, for details regarding this single component see Sections S1, S2, and S4, Supporting Information), that are coupled to a number of straight waveguides (see e.g., Figure 1a,c). Laser light sent into any one of the different straight input waveguides will only couple significantly to those rings along the light path that have a resonance close to the wavelength of the laser. At the same time, these rings will also act as connections to neighboring straight waveguides, setting up a non‐trivial interconnection topology that depends on the wavelength, on the PCM states and, for high enough input power, on the volatile nonlinear effects in silicon. Considering for example a triangular PPRRNN (i.e., one in which the MRRs linking straight waveguides form a triangular arrangement, as in Figure 1b), measurements of spectra in the linear regime, i.e., for low enough input power, show the overlapping of the resonance dips of different MRRs (Figure 1b). Because of light interference, nontrivial spectral features can arise from the coupling of multiple MRRs.

Although the MRRs are designed to be identical, each one shows a different resonance wavelength due to fabrication imperfections. While this often limits the scalability of systems based on cascaded MRRs, all the demonstrated and proposed uses of PPRRNNs in our article are not hindered by node variations due to fabrication errors, as we do not need to optimize parameters before fabrication. In this particular chip, resonances are generally red‐shifted as the MRR position is moved to the right or upward, with a smaller random shift superimposed on top of this. Thanks to this correspondence between MRR position in the spatial and in frequency domain, significantly different input wavelengths (i.e., significantly larger than the MRR resonance width) in a PPRRNN can be coupled to different groups of MRRs (see an example in Figure 1c). Operationally, each group of coupled MRRs corresponds to a different virtual network, which can operate separately and in parallel if the corresponding input wavelengths are different enough. Exploiting this property, a PPRRNN can be designed to host a few large networks comprising many coupled MRRs, or many smaller networks that can work separately and in parallel at different wavelengths, even sharing the same input ports. Moreover, many different wavelengths can excite the same group or overlapping groups of nodes, through the different quasi‐periodic resonances in a single MRR. These virtual networks are an important ingredient in the scalability properties of a PPRRNN, as they do not require additional chip area.

If the network input has high enough power, the silicon nonlinear effects in the excited MRRs can shift and change the shape of the resonance dips, enabling complex dynamic responses.^[^ ^30^, ^32^ ^]^ In particular, temporary resonance perturbations due to free carriers and thermal effects (blue and red shift respectively) provide the nonlinear activation functions of the artificial neurons but also, respectively, short and long term volatile memory. Moreover, those MRRs with a PCM cell (one in every three in each row was chosen for this work) also feature non‐volatile memory. In Figure 1d we depict the ANN diagram corresponding to the physical PPRRNN in Figure 1c, showing the main connectivity and memory elements, leaving out the dependence of the neuron response to the input wavelength. In this work we consider the optical connections (grey arrows) as instantaneous w.r.t. the dynamics of input signals and memory effects (recurrent arrows), since light propagation happens much faster. Therefore, the memory effects of the nodes are in practice applied to the equilibrium state of the purely optical network dynamics.

It should be stressed that the plastic nodes (MRRs with PCM) have less pronounced nonlinear and volatile memory effects than bare MRRs, due to the lower Q factor caused by the optical loss at the PCM cell. Therefore, in Figure 1d we neglect the weaker memory due to free carriers (blue arrows), while temperature still has a significant but reduced influence. In this section we have introduced a triangular PPRRNN in order to show how the spectrum of an increasing number of coupled nodes builds up. However, from now on, we will only consider rectangular PPRRNNs, which are more compact.

## 3 Emergent Synaptic Plasticity Enables Self‐Adaptive Non‐Volatile Weight Modifications Without External Control

In this section, we present an experimental investigation on self‐adaptation due to the emergent plasticity property in a PPRRNN, and on how it can be exploited to improve machine learning (ML) performance on a time‐series classification task, without explicitly tuning the network weights externally. In particular, as we explain below in more details, we repeatedly insert a specific waveform into our PPRRNN to induce plastic adaptation, which stops only when the network “learns” to dissipate the optical power so that the PCM cells are not significantly switched anymore by the input waveform. Therefore, the obtained non‐volatile weights configurations are the result of the complex interplay between network activity and plastic adaptation of PCM cells, and the feedback given by energy dissipation. Indeed, we show that the non‐volatile weights configuration obtained through this process strongly depends on the employed input waveform, allowing us to explore the parameter configuration along different directions without resetting the PCM cells and without additional connections to control the weights. Here, our main aim is to demonstrate that non‐volatile plasticity in our network is *rich*, *accessible* and can be *concurrent* with volatile nonlinear memory. By *rich*, we mean that multiple and significantly different non‐volatile plastic configurations can be realized by slightly different input optical waveforms. By *accessible*, we mean that these plastic configurations can be obtained using reasonable time‐dependent optical input signals (not too powerful, not too noise‐sensitive response, not too slow or fast, etc.) and affect the network output in a well‐readable way. *Concurrency* with volatile nonlinear effects means that the network is able to exploit both non‐volatile (plastic) and volatile memory at the same time, in order to carry out a task. Indeed, richness, accessibility and concurrency with volatile effects are three fundamental properties for physical plasticity in order to be practically employable for biologically plausible learning based on self‐adaptation. Here we demonstrate, for the first time to the best of our knowledge, all three attributes to be readily available in a photonic hardware.

**Figure 2:**
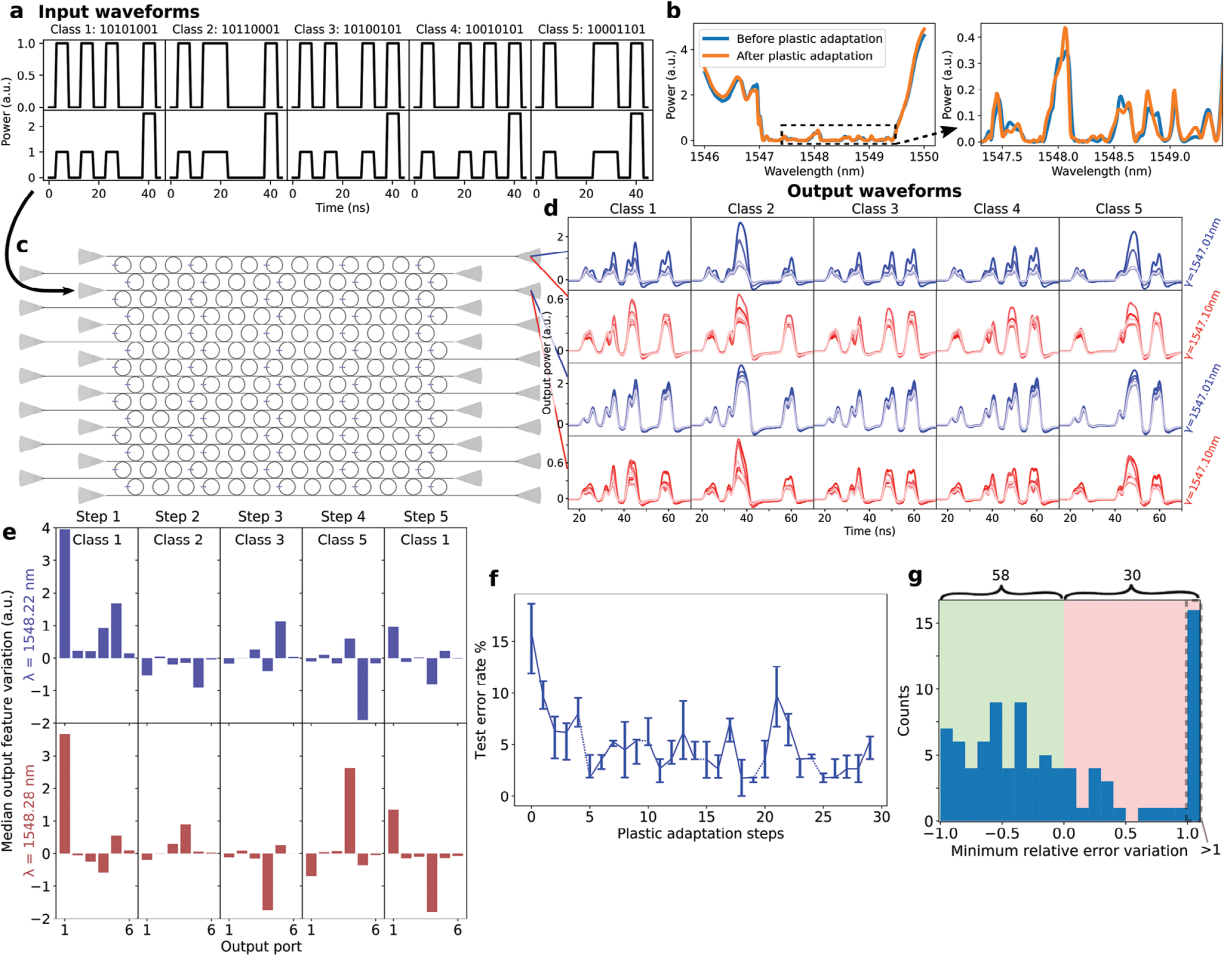
Impact of plasticity on the network response. a) Example of input waveforms from the considered five classes (columns), employed in the inference step (first row) and in the plastic adaptation (PA) step (second row). b) Example of non‐volatile spectrum modification due to the plastic adaptation of the considered PPRRNN (measured using the third input port and the third output port). c) Schematic of the considered PPRRNN. d) Examples of average output waveforms (median, 10% and 90% percentiles) for different input waveform classes (columns) at two different ports and two different wavelengths (rows). The five different shades in the plots correspond, from dark to light, to the output obtained at the beginning and after different subsequent PA steps from class 1 to 5. e) Example of variations of the median output features (last pulse energy of output waveforms) for different output ports, due to different classes of consecutive PA steps (columns) and for two input wavelengths (rows). f) Example of error rates (for the considered five‐classes waveform classification) in a PA step sequence, as a function of consecutive PA steps. g) Histogram of the minimum error rate relative variations w.r.t. the initial error, in each measured PA step sequence. This provides an idea of how often and how strongly the PA step sequences improves (negative values on the x axis) the corresponding initial ML performance.

In this section we study an example application in order to gain insight into the plasticity properties of our network, by employing a bespoke task and a dedicated optical training scheme, which allows us to study the system in a more controlled environment. For the task, we consider five classes of input waveforms, each being a different permutation of four high bits over eight bit positions in time (see upper plots in **Figure** **2**a). These waveforms have the additional constraint that, at the first and the last positions, bits are always high. Altogether, this represents a temporal frame. A single bit is 5 ns long. During the inference phase, a high bit has a relatively low peak power of around 7 mW. The pulse power is chosen so that it can trigger nonlinear volatile effects (thus the output waveforms present nonlinear distortions w.r.t. the input) but not significant non‐volatile changes. That is, dimensionality expansion can be obtained while the solid‐state phase change of the GST patches is considered negligible. Under these conditions, we can test the ML performance for a certain fixed configuration of the non‐volatile weights in the network. Figure 2d shows examples of average output waveforms corresponding to the five classes (columns), for two different wavelengths (blue for 1549.01 nm and red for 1547.10 nm) and at output ports in rows 1 and 3, with reference to the PPRRNN in Figure 2c. Here, it should be stressed that, in order to give an idea of the noise and of potential instability in the acquired network output, we plotted for each output waveform and in the same color the median and both the 10% and 90% percentiles, from a sequence of five repetitions of a certain input. The different shades of blue and red, instead, show to the output after different plastic adaptations of the network, which will be discussed later on in this section.

We employed a simple ML pipeline to evaluate the network performance for different plastic configurations: for each input waveform, we applied a linear classifier (logistic regression) on only a single value per output waveform. This value is the output energy corresponding to the last pulse of the input waveform at the considered output port. This particular choice for a small number of features makes the ML task far more difficult. Importantly, without the volatile memory and the nonlinearity provided by our PPRRNN, it is in principle impossible for the employed classifier to learn the classification, since the last transmitted pulse would be independent on the previous pulses. (For clarity, here we stress that a single classifier is applied to all the network outputs, as opposed to the ML model described in the next section, where multiple linear classifiers are applied each to a single output waveform and then combined).

In order to investigate the variations of the network response and of the corresponding ML performance due to plastic PCM weights modifications, we alternate so‐called *inference* and *plastic adaptation steps* (from now on we will shorten the latter to *PA steps*), which we now explain.

During an inference step, the waveform classes are repeatedly inserted into the PPRRNN, one after another but always separated by around 2μs to eliminate thermal memory between waveforms. The energy of the last pulse of the obtained output waveform is used as the sole feature for the linear classifier.

During a PA step, a modified version (called here *pump waveform*) of one waveform class is repeatedly inserted. In particular, a pump waveform from a given class is the same as a normal input waveform from the same class, with the only difference that its last pulse has significantly larger peak power (usually by a factor 2.5, see lower plots in Figure 2a). The enhanced power in the last pulse is chosen so that it can significantly modify the accessed plastic PCM weights in the network. On the other hand, the first three pulses of a pump waveform are meant to set the same volatile PPRRNN configuration obtained by the first three pulses in a corresponding normal waveform, so that the last enhanced pulse reaches the same nodes and output ports as the last pulse in a normal waveform. This way we aim to demonstrate *richness* of plasticity, showing that different classes of pump waveforms can modify the plastic weight configuration in different ways, related to the specific light path induced by the corresponding class of normal input waveforms. It should be specified that both inference and PA steps consist of a repetition of the waveform with a total duration of around 1 s. Therefore, given the large number of inserted waveform copies (hundreds of thousands), the plastic weight configuration is considered to have reached an equilibrium non‐volatile state after each PA step, depending on the class used in the step and also on the order of the classes used in previous PA steps. Figure 2b shows an example of non‐volatile spectrum modification due to plastic adaptation of the considered PPRRNN. Further practical details regarding the plasticity investigation and the ordering of classes during training can be found in the *Methods*, Section 7.3.

A first result we will demonstrate in this section is that different input (pump) waveforms can achieve significantly different plastic weight configurations. Therefore, we now analyze the output of our physical network, without considering any specific ML task. An example is given by Figure 2d, where the five different shades in the plots correspond, from darkest to lightest, to the average output waveforms obtained at the beginning and after different subsequent PA steps, spanning over the different waveform classes. We can notice that the output variations due to the rearrangement of internal plastic weights by PA steps are substantial and easy to distinguish. Thus, this shows that network plasticity is well *accessible* in our PPRRNN. Moreover, all the output waveforms present evident and different nonlinear distortions w.r.t. to the input shown in the first row of Figure 2a. Since each curve corresponds to measurements for a fixed nonvolatile configuration of the plastic weights, these distortions are due to volatile nonlinear effects. This demonstrates that we can achieve non‐volatile plastic adaptation *concurrently* together with volatile nonlinearity.

The output variation due to plastic adaptations (represented, as we have just discussed, by the different shades in the plots of Figure 2d) is more accurately quantified in Figure 2e, where the bar plots show the median variation of each output feature (i.e., the intensity of the last pulse) for the different output ports (on the x axis). Each plot column shows the variation due to a different PA step, in chronological order, for two wavelengths (rows). It can be easily noticed that different variations are obtained after each different PA step, implying that different plastic weight configurations are achieved in the PPRRNN. Thus, this demonstrates *richness* of network plasticity in our PPRRNN. A more detailed discussion of richness using more extensive experimental results is given in Section S3 (Supporting Information).

In the previous paragraphs we have discussed plastic effects by directly looking at the network output. Now we take a step further and see how network plasticity can be useful in ML. In particular, we repeatedly evaluate the aforementioned waveform classification task after different PA steps. Mainly, we are interested in seeing how different plastic weights configurations result in different ML performance, i.e., in different performance of the PPRRNN when employed to provide useful data representation to be fed to a linear classifier. Indeed, we will see that sequences of PA steps allow us to explore the plastic weights' configuration space so that it is likely to achieve significant ML improvements w.r.t. the initial (unadapted) state. An example of how the repetition of different PA steps can greatly improve the ML performance (regarding the classification of the five different bit patterns) is shown in Figure 2f, where the initial error rate in a PA step sequence is decreased by more than a factor 6. Remarkably, after the improvement due to the first six PA steps, the error rate stays significantly lower than its initial value for the subsequent steps. This result shows that our approach to modify the plastic weight configuration of the PPRRNN can permanently improve the linear separability of the output feature values. Importantly, this is achieved without any externally supervised weight training, but just by letting the plastic network adapt to its input, in an emergent fashion. In this way, the proposed method resembles to the way biological brains memorize and learn.

However, not every measured PA step sequence (each distinguished by a different wavelength or input port, see Table 2) resulted in such an evident and stable performance improvement. Nevertheless, we will show that they still allow one to explore the plastic weight configuration space such that ML performances are often significantly improved. In order to illustrate this, we look at how much the ML error decreases w.r.t. the initial network state, as the consequence of subsequent PA steps. For example, considering the PA step sequence evaluated in Figure 2f, we are interested in the improvement corresponding to the minimum error achieved w.r.t. the initial error value. In practice, for each measured PA step sequence, we calculate the minimum error rate variation (which is negative if there is a classification improvement) relative to the corresponding initial error rate value, where the minimum is taken over the error values achieved by all the PA steps in the sequence. We calculated this minimum (relative) error variation for each measured PA step sequence, and plotted them in a histogram (Figure 2g). It can be noticed that most of the PA step sequences allow to improve the classification performance (shown by negative values on the x axis). Importantly, the distribution does not decrease as the values on the x axis approaches –1 (which corresponds to a complete removal of the initial error thanks to the PA step sequence). This shows that strong performance improvements, as a result of a PA step sequence, are roughly as frequent as small ones.

**Table 1:** Comparison of MNIST classification accuracy experimentally demonstrated employing photonic neuromorphic hardware.

Work	System	ML approach	MNIST accuracy	On‐chip area
This work	Integrated recurrent ANN based on photonic resonators, with combination of linear classifiers applied on each time‐dependent output. With simple preprocessing (mainly downsampling).	Ensemble (chaining) of RC systems based on logistic regression. Samples: 2941 train, 2941 test, with 4x or 5x data augmentation.	98.2%	∼0.5 mm^2^
Nakajima et al. (2022)^[^ ^11^ ^]^	FPGA‐assisted fiber‐optic system implementing optoelectronic time delay RC.	Deep RC, trained with augmented direct feedback alignment (DFA).	97.80%	No integration
Mourgias‐Alexandris et al. (2022)^[^ ^40^ ^]^	CNN (in software) fed into two final photonic layers.	Standard BP on software using a noise‐aware training model.	99.3%	3 chips, at least 20 mm^2^ each
Zhou et al. (2021)^[^ ^41^ ^]^	3‐layer ANN employing large‐scale optoelectronic diffractive processing units.	Training in software using BP and adaptive training steps of the optics to adjust for the experimental error.	96.6%	No integration
Feldmann et al. (2021)^[^ ^36^ ^]^	On‐chip photonic crossbar array with PCM performing matrix‐vector multiplication for CNN acceleration and a fully connected layer in software.	Standard BP in software.	95.3%	At least 25 mm^2^
Antonik et al. (2019)^[^ ^42^ ^]^	Laser, SLM and camera providing a large‐scale optoelectronic ANN layer.	RC on extracted features (histograms of oriented gradients, in software)	98.97%	No integration
Nakajima el al. (2021)^[^ ^43^ ^]^	On‐chip recurrent, passive and coherent photonic network.	RC (spatiotemporal).	91.3%	∼32 cm^2^
Bai et al. (2023)^[^ ^44^ ^]^	CNN, where the linear part of the convolutional layer is performed by on‐chip photonic devices and circuitry. Two fully connected layers follow in software.	Standard BP and in‐situ calibration based on gradient descent control. 500 test samples.	96.6%	∼0.75 mm^2^
Zhu et al. (2022)^[^ ^45^ ^]^	2‐layer ANN, each layer based on a programmable integrated photonic network, with preprocessing.	Standard BP on software. 500 test samples.	91.4%	0.53 mm^2^
Oguz et al. (2023)^[^ ^46^ ^]^	2 convolutional layers (software), each followed by an untrained optical nonlinear mapping (via multimode fiber), with a final readout layer (software).	Gradient‐free training through the recently proposed Forward Forward Algorithm (FFA).^[^ ^10^ ^]^ Samples: 4000 train, 1000 test.	94.4%	No integration

**Table 2:** List of performed measurements considered for the plasticity investigation presented in Section 3.

Measurement session	Input port	Output ports	Classes in PA steps	Wavelength range center
1	3	1,3,5,9	2,3,4,5	1547.06 nm
2	3	1,3,5,7	5	1547.16 nm
3	2	1,2,3,4,6	1,2,3,5,1	1548.28 nm
4	1	1	1,2,3,5,1,2,3	1547.85 nm
5	2	2	6 repetitions of 1,2,3,4,5	1549.27 nm (single)
6	2	2	6 repetitions of 1,2,3,4,5	1548.12 nm (single)
7	2	2	6 repetitions of 1,2,3,4,5	1547.86 nm (single)
8	2	2	6 repetitions of 1,2,3,4,5	1547.28 nm (single)
9	2	2	6 repetitions of 1,2,3,4,5	1547.26 nm (single)
10	2	2	6 repetitions of 1,2,3,4,5	1547.19 nm (single)
11	2	2	6 repetitions of 1,2,3,4,5	1547.17 nm (single)
12	1	1	6 repetitions of 1,2,3,4,5	1548.28 nm (single)

In this section we demonstrated that the non‐volatile parameter space of our PPRRNN can be explored by using different input sequences, to reduce the network error. This type of training approach is expected to have much slower convergence w.r.t. to other approaches where credit assignment is more directly tackled. Nevertheless, plastic self‐adaptation in our PPRRNN can be potentially exploited for more sophisticated training approaches, for example by modulating a PA step with the error of the network, as we discuss in Section 5. It should be stressed that demonstrating a full training procedure that optimizes the plastic weights in the PPRRNN independently of the initial conditions of the network (e.g., given by the arrangement of the nodes' resonant wavelengths w.r.t. the input wavelength), is considered as an ambitious goal for a future work. In the next section, instead, we show that different output waveforms (that are nonlinear representations of the input) can be exploited as‐they‐are by a suitable ML procedure, achieving high accuracy in a far more complex benchmark task (handwritten digits classification).

## 4 Combining Parallel Temporal Representations For Improved Machine Learning Performance (MNIST Classification)

We will now proceed to present a more universal and relevant benchmark task, namely the ten‐class image classification problem from the MNIST dataset for handwritten digits.^[^ ^28^ ^]^ Here we employ a more practical learning scheme that needs no modification of the input waveform to enable plasticity. Additionally, to increase robustness and to better exploit the computational power and multiplexing capabilities of our hardware, we introduce a hierarchical scheme consisting of many networks, where each network is trained to improve upon the performance of the previous one. Importantly, the elements in the hierarchy do not need to be separate structures, but can be different virtual structures in the same network, realized by changing ports and wavelengths.

**Figure 3:**
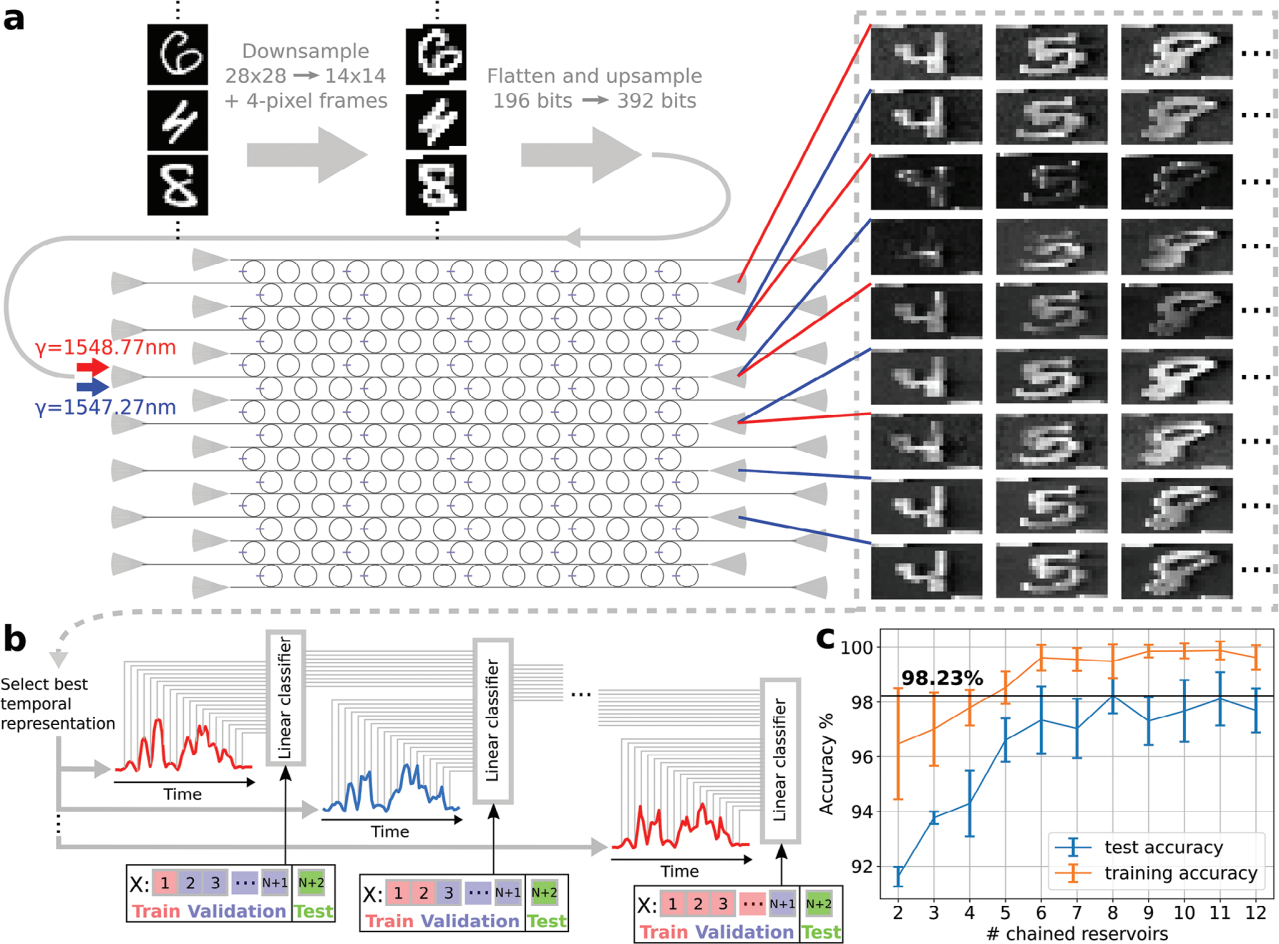
MNIST images classification combining different output representations from a PPRRNN. a) Example of images insertion into a PPRRNN and of the resulting output representations (in this figure these are spatially rearranged to enable visual intuition, but in truth each is a 1D time‐dependent output). After downsampling (see Section 7.4 for more details), flattened images are inserted into a single port (grating coupler on the left) using two wavelengths. Nine different nonlinear representations of the flattened images are then obtained from different output ports and input wavelengths. b) Linear ML classifiers are applied to the output representations and combined using a *chaining* ensemble method. Here, each output representation and classifier pair is considered as an RC system, representing the reservoir and the readout layer respectively. A high‐level diagram is provided in Section S4 (Supporting Information). c) Classification accuracy as a function of the number of chained representations and classifiers. The best average test accuracy obtained is 98.2%.

We start by flattening each image in the MNIST dataset and insert it as a single time‐dependent input that can trigger volatile memory and non‐volatile self‐adaptation concurrently in a rectangular PPRRNN. Very little preprocessing was employed, motivated exclusively by limitations of the experimental setup (see Experimental Section 7.4 for more details). As discussed in the previous section, different nonlinear output representations of the input waveform can be obtained in parallel from different physical output ports and for different input wavelengths. Each representation is a waveform (a time‐dependent 1D signal) that can be reassembled into a corresponding flattened image of *n* pixels (see examples of MNIST images output representations in **Figure** **3a**). Differently from the application of kernels in a convolutional neural network (CNN), the generation of these representations is not the outcome of an external learning algorithm, but it depends on the emergent plastic and volatile properties of the PPRRNN and on how the input is inserted (power, wavelength, bitrate). Here we will show that an ensemble of linear classifiers (multi‐class logistic regressors) can learn how to synthesize and combine the information unveiled in these representations, in order to greatly improve classification performance, while still having a backpropagation‐free lightweight machine learning (ML) pipeline.

In particular, we considered each parallel PPRRNN's representation of the input samples as the output of a stand‐alone *reservoir*, according to the *reservoir computing* (RC) framework,^[^ ^37^, ^38^ ^]^ thus treating our PPRRNN as a collection of coupled reservoirs working in parallel. RC is a hardware‐friendly ML approach where only a linear model (a single‐layer ANN) is trained, and is applied to the output of a fixed nonlinear dynamical system (the reservoir, e.g., a recurrent neural network with fixed synaptic weights), that is in turn excited by a time‐dependent input. Normally, a reservoir has only volatile memory, while our PPRRNN also features non‐volatile memory. But, since in this section we do not formally train the PPRRNN, although it plastically adapts to its input, for simplicity's sake we still refer to its representations as *reservoirs*. Following the RC scheme, we applied and trained a linear classifier (in software) on each PPRRNN's representation, forming an RC system per representation. Each single RC system alone, though, is a rather weak classifier (the accuracy does not exceed 88%, which is close to the accuracy of a linear classifier directly applied to the input, without any neuromorphic hardware). However, we could assemble a much stronger classifier by combining the RC systems together in a special way (see Figure 3b; Section S4, Supporting Information; Section 7.4 in Experimental Section for technical details), such that each RC system is be trained to correct the errors made by the previous reservoir. This scheme exploits the PPRRNN's capability of efficiently producing several different nonlinear representation of a temporal input at the same time, leaving to the electronics only the linear models, which are easy to train and computationally cheap. Importantly, the training procedure does not require gradients, is backpropagation‐free and provides an example of how the PPRRNN can be employed for powerful biologically‐plausible neuromorphic computing.

Figure 3c shows the obtained test and training accuracy averaged over the cross‐validation loop, as a function of the number *N* of classifiers chained together. The error bars represent the standard deviation of the cross‐validation accuracy. It can be noticed that our chaining method significantly improves the classification accuracy and reduces overfitting as the number of chained representations increases, until the improvement saturates. We obtained a maximum average accuracy of 98.2% from a chain of length 8. Importantly, a much lower maximum accuracy (around 92%) was reached if we employ a more straightforward method of combining the representations, namely stacking all the features together to obtain a large spatio‐temporal representation on which the readout was trained. This is probably caused by overfitting because of the larger number of features in a single training. On the other hand, we believe significant improvement in maximum accuracy for both methods could still be achieved just by measuring more samples to employ for training.

Finally, we compare our best average classification accuracy with the ones experimentally demonstrated in other works about photonic neuromorphic hardware (see **Table** **1**). We achieved a high accuracy compared to other works, demonstrating a high computational power and stability of the plastic spatio‐temporal representations produced by our photonic network. Moreover, the two works outperforming our accuracy level rely heavily on powerful feature extraction performed in software.

In fairness, the aim of the compared works is often to improve also on performance parameters other than accuracy, like energy efficiency or throughput per footprint area. However, these other parameters are usually expressed in terms of number of multiply‐accumulate operations (MACs) that can be trained and performed by an ANN. Since we employ a hardware‐based dynamical system approach, it makes little sense to directly compare, e.g., energy efficiency in terms of MACs/J, because the operations in our recurrent neural network are not externally programmable, although they can be reconfigured via plastic self‐adaptation. Nevertheless, in order to give a quantitative idea of the computational throughput per chip area and of the energy efficiency of a PPRRNN, we approximately estimated 10^15^ (MACs+NLOs)/s/mm^2^ and 5 × 10^15^ (MACs+NLOs)/J respectively (see Section 7.5 under Experimental Section for details on this estimation). The units for these quantities are similar to the usual energy and aerial efficiency estimations (MACs/s/mm^2^ and MACs/J respectively), but imply that each MAC operation is also accompanied by a nonlinear operation (NLO), since we work with nonlinear neurons. It should be stressed that the nonlinearity in each node in principle enhances the computational power of our system w.r.t. to photonic linear accelerators, although our synaptic weights cannot be precisely and individually programmed. Moreover, in our estimation we neglected the operations happening in the hidden recurrent layers in our PPRRNN, so as to make it somehow more comparable with linear accelerators, which perform one single layer of weighted connections at a time. Even so, the estimated computational throughputs and efficiencies for our PPRRNN are well beyond those of photonic neuromorphic hardware where synaptic weights are precisely programmable.^[^ ^18^, ^39^ ^]^ Furthermore, it should be noticed that it is straightforward to significantly increase aerial and energy efficiencies by using MRRs with lower radii, higher Q factors and shorter GST patches. Further details regarding energy consumption, footprint and throughput of the employed PPRRNNs are discussed in Experimental Section, Section 7.5.

## 5 Discussion

### 5.1 Mechanisms and Properties for High Scalability

In Section 3, we have shown that different plastic weight configurations could be achieved in a PPRRNN by insertion of different time‐dependent optical signals. Moreover, these non‐volatile modifications could often improve the system performance on a simple time‐series classification task. Since plastic adaptation is a key mechanism for learning and memory in biological brains,^[^ ^12^ ^]^ learning with physical plasticity is an extremely relevant research direction for the development of neuromorphic computing. Indeed, developing a physical platform where dynamics, nonlinearity, volatile and non‐volatile memory coexist in complex scalable networks is important in order to provide a physical and experimental underpinning to such a research effort, which is nowadays mainly limited to simulations or entirely externally implemented learning rules.^[^ ^6^ ^]^ In fact, simulating large‐scale dynamical systems is arduous and requires large computational resources and simplistic approximations. For instance, approximating or neglecting constraints or richness of response found in physical systems, might prevent the discovery of important learning mechanisms in biological neural networks.

In this section we present the main aspects and properties enabling scalability (in terms of network computational power and size) of the proposed neuromorphic computing approach. Here we would like to highlight the differences between our approach, without externally tunable parameters and based on linear classifiers like in reservoir computing (RC), and the more common one, based on employing backpropagation (BP) on a simulated version of the ANN, whose trained parameters can be transferred to the hardware network via external tuning and correction of hardware non‐idealities. In our approach we have reduced control and configurability, and in particular we give up the possibility to accurately predict (before fabrication) and simulate the response of our network, which would have allowed us to estimate the gradient of the cost function and exploit powerful training methods based on BP. In turn, we gain in complexity (having a relatively large number of highly dynamical and nonlinear nodes with multi‐scale memory), in robustness to fabrication errors and in low footprint. Moreover, by introducing the all‐optical plasticity given by PCM cells in our network, we aim to mitigate the loss in control by potentially allowing for a more biologically plausible way to optimize network parameters, through plasticity and emergent self‐adaptation.

#### Synaptic weight modification without external connections and credit assignment

As mentioned before, today state‐of‐the‐art ANNs are trained using BP, which is considered not biologically plausible.^[^ ^6^, ^7^, ^8^ ^]^ BP requires full observability of the neuron states and full tunability of parameters, such as synaptic weights. In practice, where neuromorphic hardware is concerned, this usually requires physical connections in order to observe states and to update weights, so as to apply a training algorithm that runs on an external computer. However, this obviously undermines the scalability of physical ANNs, preventing the use of a large number of neurons and synapses. In this work, instead, plastic weights are modified in a more biologically plausible way through self‐adaptation, by exciting the input ports of the network, without requiring dedicated connections. An additional potential advantage of this approach is that, since the signal modifying the plastic weights is inserted at the network input and travels through the normal network connections, the updating of the plastic weights naturally contains information regarding the state of previous nodes and links along the activation path. For example, if a certain ML sample (in our case corresponding to a time‐dependent input) is associated with a high inference error, it is possible to selectively modify the self‐adapting weights responsible for the low performance of that specific sample by simply letting the network adapt to the sample and, if needed, by increasing the power of part of the input waveform, as it was done in Section 3. This would allow us to tackle the credit assignment problem without any backward pass nor internal gradient information. Another possible training approach would be to modulate the input waveform power (or part of it) with the network error, so as to favor convergence to equilibrium states in response to low error levels.

#### Dynamics‐enabled cascadability

Cascadability of nodes, and also of plastic connections in our case, is critical for scalability of ANNs. However, it is often difficult to achieve in hardware implementations (especially in photonics) without employing a large number of amplification stages or alternative signal sources to compensate for propagation losses, and this may strongly limit scalability. This problem is mitigated when suitably using silicon MRRs as a dynamic node. Indeed, let us consider a PPRRNN (see Figure 2c) with rows containing many MRRs in series, assuming for simplicity that the resonant wavelengths are aligned. A non‐resonant optical input pulse will reach the corresponding direct output port with potentially negligible energy loss. Instead, a resonant input pulse will be totally or partially absorbed by the first encountered node. However, if the nodes are suitably designed and if the pulse carries enough power, the resonance wavelength of the first node will be red‐shifted out of the way by heating due to optical absorption. Therefore, a subsequent pulse can reach the second MRR, whose resonance can be shifted as well, and so on. This way, a detectable signal can reach an output port even when the transmission in the linear regime through the original optical path is very low.

#### Parallelism and network expansion via wavelength division multiplexing

As discussed in Section 2, even if each node has only four physical connections, these can convey several different signals in parallel and independently by means of WDM, thus greatly expanding the number of network connections, as well as the number of input and output ports. Thanks to the quasi‐periodic resonances in the spectrum of a MRR, several different signals at different wavelengths can nonlinearly interact through the silicon nonlinear effects in the optical cavity. Indeed, a powerful enough resonant pulse will simultaneously modify (either temporally or permanently) all the resonances in the MRR spectrum, thus changing the way pulses at other wavelengths excite (or are transmitted by) the neuron. In practice, this mechanism expands the fan‐in and fan‐out properties of both the artificial neurons and the plastic nodes, whose activation can be achieved by the total power carried by pulses at different times, at different wavelengths and at different physical connections (in the latter case, optical interference comes into play as well).

#### Multi‐timescale computation

Nowadays, a major challenge in the development of neuromorphic computing platforms for edge computing is the need to match the timescales of the computing system with the ones of the input information, which may depend on, e.g., the type of physical quantities targeted in smart sensing applications.^[^ ^47^ ^]^ The PPRRNN proposed here presents dynamic responses with multiple timescales, which can potentially be expanded or controlled. In particular, the fastest timescale is given by the travel time of light signals through the network, considering also that MRRs accumulate resonant light with typical transient times of tens of picoseconds. This timescale can be controlled and extended by choosing the Q factors of the MMRs, or, more effectively, by introducing optical delay lines in the photonic circuit.^[^ ^48^ ^]^ The second fastest timescale is given by silicon nonlinear effects related to free carrier concentration in ring waveguides, of which time constants can range from a few to tens of nanoseconds. These can also be controlled by applying a suitable p‐n junction to the ring waveguide.^[^ ^49^ ^]^ The slowest timescale is provided by the thermo‐optic effect either in ring waveguides or in the PCM layer, with characteristic times of hundreds of nanoseconds. Next, the thermal timescale is mainly governed by the temperature dissipation, which can potentially be tuned by design of the photonic circuitry. Interestingly, the combination of effects due to free carriers and temperature can generate self‐sustained dynamics in MRRs (self‐pulsing) capable of complex and chaotic behavior.^[^ ^32^ ^]^ By suitably tuning the excitation parameters, the decay time of this type of network response can in principle be controlled and extended to slower timescales. Finally, the non‐volatile all‐optical memory introduced by PCM, allows our PPRRNNs to couple signals inserted at arbitrarily distant times enabling, for suitable types of input excitation, timescale‐invariant computation.

#### High‐throughput generation and large choice of data representations within a low footprint

As discussed in Section 4, a large number of different nonlinear representations of an input time series can be achieved by a PPRRNN with a relatively low footprint (e.g., 0.5 mm^2^), by considering different input and output ports, wavelengths, plastic weights configurations and even input signal parameters (such as bitrate and power). Therefore, once the circuit is fabricated, it is possible to explore its response so as to find the parameters providing representations suitable for the considered application, e.g. employing an RC‐based approach as we did in Section 4. Moreover, several representations can be obtained in parallel at different output ports. Furthermore, the number of parallel representations can be multiplied by considering different input ports and different enough wavelengths (see Section 4). This possibility in principle allows for the generation of hundreds of different representations in parallel within footprints of the order of 1 mm^2^, enabling high‐throughput neuromorphic computing.

### 5.2 Relation to Biologically Plausible In Situ Training Methods

Finally, let us briefly discuss the links between our photonic neuromorphic system and two inspiring training approaches aiming at biological plausibility and simplicity of implementation in hardware. In ref. [10], a surprisingly powerful learning procedure (called *Forward–Forward* algorithm, or FF algorithm) is presented, which replaces the forward and backward passes of BP‐based training by two forward passes, with the only difference being the inserted data. This makes the algorithm more biologically plausible and eliminates the BP requirement of accurately knowing all the operations performed by the network. Therefore, the FF algorithm can be implemented in hardware implementations of neural networks, where internal operations are mostly unknown due to the variability arising from fabrication errors and due to complex nonlinear responses of the nodes. Similarly, in this work (Section 3), there is no backward pass and we modify the network parameters directly by inserting specific input signals, leveraging intrinsic physical plasticity rather than an external learning rule. Another similarity is that in both cases several linear classifiers are trained and then combined (see Section 4), although in a different way. In the cited article, it is also stressed that *mortal computation*, i.e., computation learned by non‐reproducible hardware like the one here presented, may generally allow for higher energy efficiency and lower fabrication costs.

A second relevant work is ref. [11], where a hardware‐friendly augmented version of *direct feedback alignment* (DFA, see ref. [50]) is presented. DFA already takes a big step toward biological plausibility and on‐hardware implementability, by removing the need for the knowledge of the full network gradient in the learning rule and by requiring the output network error as the only non‐local information. In the augmented DFA method presented in ref. [11], the network knowledge required by the learning rule is further reduced, by replacing the differential of the activation function with an arbitrary nonlinear function. This results in a hardware friendly deep learning approach approximating BP, demonstrated both with software examples and within optoelectronic hardware (deep reservoir computer). Interestingly, high performance is obtained for different benchmark tasks. Importantly, the PPRRNN here proposed can be in principle trained employing this augmented DFA approach, by using the input signal to convey the output overall error and thus letting the plastic weights adapt to the error information.

## 6 Conclusion 

We presented an experimental investigation of a new type of integrated photonic artificial neural network based on silicon ring resonators and phase change material cells (GST). We demonstrate, for the first time, complex nonlinear behavior and multi‐scale volatile memory (provided by silicon nonlinear effects), concurrently with all‐optical non‐volatile memory (provided by GST cells).

We investigated how our network can plastically adapt to different input temporal sequences, thanks to the non‐volatile all‐optical memory introduced by the phase change material cells. This adaptation happens in an emergent way, and does not rely on external control. As part of this study on plasticity mechanisms, we investigate a simple but highly nonlinear machine learning problem, consisting of the classification of five different temporal sequences of four optical pulses. We applied a novel method to modify the network internal weights exclusively via different input signals (leveraging plastic adaptation) and we showed that these modifications often significantly improve the machine learning performances compared to initial configurations.

Moreover, in order to evaluate how powerful is the presented system in practice, we tackled a benchmark machine learning task, namely the classification of images from the MNIST dataset. Each image was inserted in the photonic network as a temporal sequence. The employed ML model does not require backprogation and consists in combining several linear classifiers (through the *chaining* ensemble method) applied to different parallel outputs of our neuromorphic hardware, where each output provides a different nonlinear representation of the input image. We achieved a surprisingly high maximum average accuracy of 98.2% and we compared it with the results from other recent works about experimental neuromorphic computing with photonics.

Finally, we discussed some properties and mechanisms enabling scalability of the proposed photonic integrated network compared to other more conventional neuromorphic computing systems, designed to be trained externally, usually via BP.

These results lay the groundwork for the application of biologically plausible and hardware‐friendly training approaches (potentially inspired by e.g., refs. [10, 11]), by exploiting the emergent plasticity property and thus without explicitly tuning the network weights. This type of training approach is particularly interesting for neuromorphic computing research, since biological brains learn and memorize by means of plastic adaptation. This allows to forego additional external connections used to tune the network parameters, as is, instead, usually required by the training of today's neuromorphic systems. Such a possibility could greatly increase the scalability of hardware ANNs, since it would allow to employ extremely complex physical systems with a large number of nodes and plastic connections, without the need to externally access every synaptic weight and neuron.

## 7 Experimental Section

### 7.1 Experimental Setup

**Figure 4:**
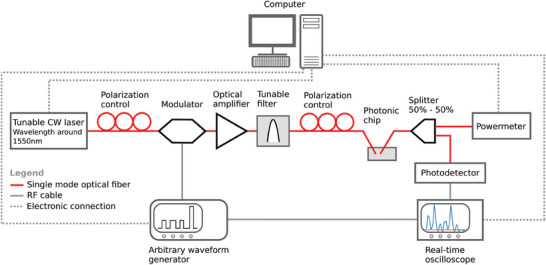
Experimental setup.

A setup (see **Figure** **4**) capable of generating a time‐dependent optical signal (max. bandwidth around 300 MHz) was employed, inserting it into a photonic integrated circuit and acquiring the response. The output of a Santec TSL‐550 tunbale laser was modulated by an X‐blue 40 GHz modulator controlled by an arbitrary waveform generator (AWG) (Moku:Lab). The signal was then amplified by an EDFA (Keopsys) and filtered with a band‐pass filter centered on the laser wavelength. The clean and modulated optical signal was coupled into and out of the photonic chip by means of fiber grating couplers. The output of the integrated circuit was split so that roughly half of the power would reach a power meter measuring the average light power, used to estimate the optical power coupled into the chip. The other output of the splitter was measured by a fast photodetector (Thorlabs balanced photodetector 1.6 GHz), whose RF electric output was acquired by an oscilloscope (Keysight Infiniivision 3000 X‐Series). A Python algorithm was running on a PC to synchronize the operations of the tunable laser (controlling power and wavelength), the AWG (controlling the type of generated waveforms), the oscilloscope and the power meter (used as reference to calculate the on‐chip optical power).

### 7.2 Design and Fabrication

The photonic circuitry was fabricated through e‐beam technology using shallow‐etched waveguides. The considered MRRs have a radius of 15μm, a coupling gap of 350 nm and a GST patch covering a section of the ring resonator of 1μm long. On the same chip, within around 63 mm^2^, 120 PPRRNNs with different topologies were designed and fabricated, number of nodes, sparsity of GST cells and coupling gap of the MRRs.

### 7.3 Practical Details of Plasticity Investigation

With reference to Section 3, by alternating inference and PA steps, the investigation was focused on how different classes of pump waveforms could achieve different non‐volatile weight configurations, and how this would impact the ML performance. In particular, once selecting an input port in the investigated PPRRNN, the focus shifted to identifying a wavelength range where significant waveform distortions would appear at the direct output (i.e., the output port that is directly connected to the input port by a straight waveguide). Then, the first inference step was performed, changing the input wavelength with 21 steps of 0.005 nm, thus spanning over a total range of 0.1 nm. This measurement was repeated for each output port and resulted in a well‐readable signal, each time acquiring between 70 and 80 waveforms per class. Therefore, an inference step provides 21 ML datasets, one for each wavelength, used to train and test the logistic regression. Moreover, we have as many ML features as the number of measured output ports. The first inference step provides a performance estimation of the considered network part (given by input port and wavelength range) with the initial non‐volatile weight configuration. Afterward, a subsequent PA step (usually of the first waveform class) modifies the non‐volatile weights configuration, which is then evaluated through a second inference step, and so on. Additionally, two nested cross‐validation loops were employed: the inner one to optimize the L2 penalty, the outer to test the trained model on all the available data.

The plotted data in Figure 2a was obtained using the third input port on the left (with reference to Figure 2c) and reading the signals at the output ports number 1, 2, 3, 4, 6, 8 on the right. The complete list of performed measurement sessions can be found in **Table** **2**.

### 7.4 Machine Learning, Preprocessing and Measurement Aspects

Regarding the MNIST classification task described in Section 4, the presented results are obtained considering 12 input‐output configurations in the network shown in Figure 3a:

1. Three representations using the fifth input port (counting the left grating couplers from top to bottom) and input wavelength λ = 1548.14 nm, at 3rd, 5th, and 13th output ports (right grating couplers from top to bottom).
2. Five representations using the sixth input port with λ = 1547.27 nm, at output port numbers 4, 6, 8, 10, and 12.
3. Four representations using again the sixth input port but with λ = 1548.77 nm, at output port numbers 2, 4, 6, and 8.

Because of limitations exclusively due to instrumentation (memory and speed of electronics to generate input waveforms and to acquire output waveforms), we employed a subset of 2941 images with balanced classes. Each image was inserted into the PPRRNN four or five times, thus performing data augmentation to improve the learning of experimental noise by the training algorithm, reaching a total sample number of 13466. Due to the same setup limitations, we downsampled the images from 28 × 28 to 14 × 14 pixels, employing the maximum over adjacent squares composed of four neighboring pixels (Figure 3a), in order to reduce the information loss due to downsampling. Each image was then flattened to a 1D array, upsampled with a factor of 2 using linear interpolation and inserted into the PPRRNN as a single waveform. It should be stressed that the preprocessing is not meant to make the ML task easier (on the contrary, it probably makes it harder because of loss of information due to downsampling) and, apart from the flattening, it should be avoided if the instrumentation allows. A bit duration of 4 ns was employed, with each flattened image temporally separated from the next by a no‐signal period of around 2μs, in order to avoid temporal cross‐talk due to thermo‐optic effects.

The MNIST class label (i.e., the ground truth) corresponding to each output waveform was encoded in the time distance between the current waveform and the next generated one. This way the class labels could be retrieved directly from the output of the PPRRNN, while ensuring that the label information could not be retrieved by the readout linear classifiers. In particular, the time distance between each generated input waveform was set to 2μs+24ns× class label (from 0 to 9, labeling the previous waveform). The output waveforms are extracted from the acquired data by detecting the presence of a signal above the noise floor, which usually corresponds to the beginning of the insertion of the input waveform, since the light delay is negligible. In those cases where this was not true, because of low transmission of the full waveform or of its initial part through the network, the output waveform was discarded.

In Section 4, the results obtained considering the 12 input‐output configurations in the network shown in Figure 3a are presented. Lets first explain the ML pipeline (Figure 3b), which consists of building a combination (chain) of linear classifiers, each applied to a different output representation. The chain is automatically built by a greedy algorithm, which adds one classifier to the chain at each step, trying to correct the mistakes made by the previous classifiers. Assume *N* output nonlinear representations were measured for each input image. Then, the available ML dataset was splitted into *N* + 2 folds with an approximately equal number of samples per class. Keep the last fold apart to use it at the very end for testing. In order to select the best stand‐alone output representation, which will be the first in our hierarchical chain, an individual linear classifier was applied to the samples of each output representation and train it on fold 1. In fact, this is analogous to training *N* reservoir computing (RC) systems,^[^ ^37^, ^38^ ^]^ since each representation could be considered as the outcome of a different untrained recurrent neural network with *n* temporal outputs. Subsequently, the best performing classifier was selected, by evaluation of a validation set containing folds 2 to *N* + 1 in the dataset. In general, each single output classifier could not outperform the baseline of directly applying a linear classifier to the input waveform (∼86% accuracy in our experiment, 88% in software^[^ ^51^ ^]^). This is indeed expected, because each representation is neither optimized to improve accuracy, nor has a sufficiently high dimensionality compared to the input, which is required for traditional RC.

In the second step of the pipeline, an ensemble of two classifiers was built by employing the *chaining* method.^[^ ^52^ ^]^ Recall that the output of the first classifier consists of ten numbers, each being the estimated probability of belonging to one of the classes. Then, a second classifier was trained on these ten numbers, combined with the samples of another representation from a different (virtual) network (Figure 3b). This linear classifier was trained on folds 1 and 2 of the dataset and its performance validated on folds from 3 to *N* + 1. This way, the second linear classifier in the chain focuses especially on correcting the errors made by the first classifier (which was trained on fold 1) in generalizing over fold 2. Therefore, this method aims to progressively improve the computational power of the ensemble of the classifiers, while reducing overfitting. Among all the *N* − 1 possible 2‐reservoirs chaining combinations, we select the one with highest validation accuracy.

Afterward, starting from the selected chain of two classifiers, we repeat the process so as to select the best three‐classifier chain, and so on until we obtain a chain of length *N*, trained on folds from 1 to *N* and validated on fold *N* + 1. The resulting validation score was employed to optimize the regularization strength of the L2 penalty in the training of the last linear classifier in the chain. Finally, the test accuracy of the obtained *N*‐reservoir chain was estimated using the unseen fold *N* + 2. This whole chain formation was repeated *N* + 2 times, each time using a different fold to select the first classifier, so as to perform a *k‐fold cross‐validation*, where *k* = *N* + 2. This way, the ML pipeline was evaluated on all the available data, in order to maximize the precision of our test accuracy evaluation.

In the experiments, the output representations at different wavelengths and physical ports were acquired sequentially, one after another. However, these results were considered to be a good approximation of a truly parallel measurement, where many photodetectors were employed at the same time, together with filters to separate the different wavelengths. Indeed, the three considered wavelengths were distant enough from each others so that they cannot be significantly coupled by the nonlinear response of the MRRs. Moreover, since the sample insertion was repeated a large number of times (much larger even than the measured repetitions) it is believed that plastic changes have reached an equilibrium by the time they are measured, allowing repeatability. Indeed, if significant changes over time were to occur during the repetitions, the classification task presented to the linear classifier would artificially become more difficult to carry out, thus limiting the achievable accuracy.

### 7.5 Energy, Footprint and Throughput of the Proposed Integrated Photonic Network

In this work, a peak on‐chip power (for a single input wavelength) of around 21 mW was employed for a fully white input pixels, and a power of around 1 mW for a black pixel. This corresponds to an on‐chip input energy per white and black pixel respectively of around 84 pJ and 4 pJ. An upper estimate for the average on‐chip energy per image is 17 nJ, which is found by assuming half the pixels to be completely white and the other half completely black: 17 nJ ≈196 × 84 pJ + 196 × 4 pJ. Regarding the on‐chip footprint, the PPRRNN considered in this section takes up around 0.5 mm^2^, providing seven physical output ports on the right side and six on the left side (even though the number of ports is doubled if the counterpropagating field is strong enough to be readable). Therefore, it could be estimated that the PPRRNN could potentially provide at least 13 nonlinear representations per mm^2^ per wavelength. A large number of representations (hundreds or even thousands) could be generated on a single chip by employing several wavelengths at the same time and considering larger or multiple PPRRNNs. In practice though, one should consider the feasibility and the impact of having many input and output optical connections on the same chip, of separating several wavelengths at the output, of employing a large number of photodetectors, of managing thermal cross‐talk, etc. However, being able to generate a large number of representations on a small chip area can be advantageous even if the representations are not read out all at the same time. Indeed, the achievable representations could be explored by automatic measurements even one by one, so as to select the best few.

Finally, here it is explained that how the approximated aerial and energy efficiency of a PPRRNN is estimated in terms of multiply‐accumulate operations (MACs) plus nonlinear operations (NLOs), namely 10^15^ (MACs+NLOs)/s/mm^2^ and 5 × 10^15^ (MACs+NLOs)/J, which are reported in Section 4. First, as the demonstrations presented in this paper, lets consider the use of free‐carrier based nonlinearity to achieve the activation function of the photonic neurons, and the temperature‐based nonlinearity as volatile memory which can integrate several neuron activations through time. A realistic case is that 2 ns input temporal resolution was used, a time duration sufficient for free‐carriers to provide strong nonlinearity, and an integration time due to the thermal memory of around 200 ns. Therefore, considering only one input port connected to one output port in a PPRRNN, each 2 ns present at the output is the result of a nonlinear integration of the input inserted in the previous 200 ns. Thus 100 MACs+NLOs operations were performed each 2 ns, considering only the time dimension. Equivalently, this system could be seen as two connected neuron layers (input and output) in the time domain, each comprising 100 neurons, neglecting for simplicity the recurrent operations in the hidden node layers. The number of MACs+NLOs was found by multiplying the dimension of the two layers, which gives 10^4^ (MACs+NLOs) each 200 ns. Moreover, in a PPRRNN fitting a 0.5 mm^2^ chip area, it is realistic to have at least ten physical input ports connected to other ten output physical ports, hence we obtain an input and an output layers of 10^3^ neurons each, achieving 10^6^ MACs+NLOs per 200 ns and per 0.5 mm^2^, covering both the time and spatial dimensions. Furthermore, in such a PPRRNN we can in principle employ at least 10 input wavelength channels per physical port, so that they are interconnected by the network activity, thus further expanding both the input and output dimensions from 10^3^ to 10^4^ each, achieving a total throughput per unit area of 10^15^(MACs+NLOs)/s/mm^2^. Regarding the energy efficiency, we consider a realistic input power of 10 mW per input physical port, yielding a power consumption of around 200 mW/mm^2^. Dividing the throughput per area by this quantity, we finally find a power efficiency of 5 × 10^15^ (MACs+NLOs)/J.

## Conflict of Interest

The authors declare no conflict of interest.

## Author Contributions

A.L. and P.B. conceived the experiment; A.L. designed the integrated circuits (with help from S.A. and F.B.P.), carried out the measurements, processed and analyzed the data under the supervision of P.B.; F.B.P. fabricated the integrated circuits under the supervision of W.H.P.P.; S.A. performed the deposition of the GST material under the supervision of H.B.; C.D.W. supervised device modeling and coordinated the interinstitutional collaboration; All authors wrote the manuscript together.

## Supporting information

Supporting Information

## Data Availability

The data that support the findings of this study are available from the corresponding author upon reasonable request.
