# Supplementary material for: Emergent Self‐Adaptation in an Integrated Photonic Neural Network for Backpropagation‐Free Learning
Source: Adv Sci (Weinh). 2024 Nov 20;12(2):2404920. doi: 10.1002/advs.202404920 (PMC11906216; doi:10.1002/advs.202404920)
Supplement: Supplementary file 1 — Supporting Information [file ADVS-12-2404920-s001.pdf]

ADVANCED SCIENCE
Open Access

Supporting Information

for *Adv. Sci.*, DOI 10.1002/advs.202404920

Emergent Self-Adaptation in an Integrated Photonic Neural Network for Backpropagation-Free Learning

Alessio Lugnan, Samarth Aggarwal, Frank Brückerhoff-Plückelmann, C. David Wright, Wolfram H. P. Pernice, Harish Bhaskaran and Peter Bienstman**

SUPPLEMENTARY MATERIAL: EMERGENT SELF-ADAPTATION IN AN INTEGRATED PHOTONIC NEURAL NETWORK FOR BACKPROPAGATION-FREE LEARNING

Alessio Lugnan
Photonics Research Group
Ghent University - imec
Ghent 9052, Belgium
alessio.lugnan.1@unitn.it

Samarth Aggarwal
Department of Materials
University of Oxford
Parks Road, Oxford OX1 3PH, UK

Frank Brückerhoff-Plückelmann
Department of Physics, CeNTech
University of Münster
Heisenbergstraße 11, 48149 Münster, Germany

C. David Wright
Department of Engineering
University of Exeter
Exeter EX4 4QF, UK

Wolfram H. P. Pernice
Department of Physics, CeNTech
University of Münster
Heisenbergstraße 11, 48149 Münster, Germany

Harish Bhaskaran
Department of Materials
University of Oxford
Parks Road, Oxford OX1 3PH, UK

Peter Bienstman
Photonics Research Group
Ghent University - imec
Ghent 9052, Belgium

1 Function and characterization of a single plastic node

In this supplementary section we discuss the function and present the experimental characterization of an integrated plastic node, namely a silicon microring resonator (MRR) with phase change material (PCM) cell (see Figure S1 a, b), which was used as a building block in photonic plastic networks discussed in the main text. First of all, the main function of the plastic node, as we employ it in this work, is to split the input light between its two output ports, with a ratio that mostly depends on the PCM solid state (i.e. on the non-volatile memory state) and on the input laser wavelength. This effect is shown by the spectra of the plastic node corresponding to different PCM states, displayed in Figure S1 d and e, and discussed below in this section. Each network node, however, is a complex physical system. Therefore, for a full theoretical description, numerical models and simulation results, we refer the reader to previous works [1, 2]. Moreover, here we provide a more formal description of the single node operation in Section 4.

A MRR is resonant only to wavelengths that can fit an integer number of times along the ring waveguide, so that the light travelling in the ring interferes constructively with itself after a round trip. If this condition is met, the input light accumulates in the ring and part of it is redirected to the *drop* port. Otherwise, for wavelengths far from the resonant condition, most of the input signal continues its travel along the straight waveguide (to the *through* port), approximately undisturbed by the presence of the resonator (see the MRR transmission spectrum in Figure S1 c). Generally, with reference to Figure S1 a, depending on the wavelength of an input optical signal at the *in* or *add* port, and depending on the GST memory state (i.e. amorphization level), different fractions of the input light will be led to the *through* port, redirected to the *drop* port, or absorbed by the ring waveguide (which also comprises a PCM cell). The latter implies heating and increase in free carrier concentration of the resonator, with consequent volatile modification of the

resonance properties, such as the resonant wavelengths. Furthermore, light absorption and consequent heating of the GST cell, if intense enough, triggers modifications to the non-volatile memory state. It should be also noticed that if light with the same wavelength enters through the two input ports at the same time, optical interference of overlapping beams will come into play as well.

In order to evaluate energy efficiency and contrast of memory operations (considering both GST amorphization and recrystallization), we sent a sequence of short optical pulses into the MRR input port, so as to change the solid-state phase of the GST cell. In particular, a single optical pulse of 10 ns duration was employed for GST amorphization, while for recrystallization we used a sequence of 100 pulses with the same duration and lower power, each separated by time intervals of 10 ns, with total length of 2 µs. We did so, instead of using the double-step pulse employed in [3], so as to demonstrate that the repetition of the same pulse shape used for amorphization can reverse the memory state as well. This is key to achieve a plastic behaviour of the node, whose memory states need to be fully accessible by employing the same input signal shape.

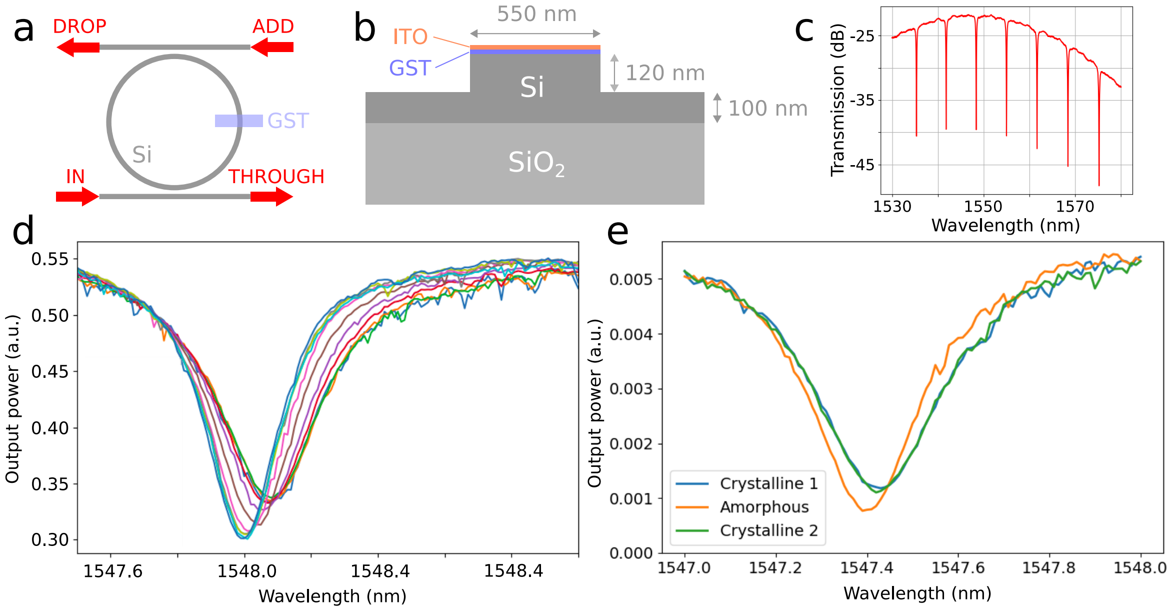

Figure S1: **All-optical plastic node.** **a** Schematic of the considered plastic node, consisting of a silicon add-drop MRR with a PCM cell of 1 µm **b** Schematic of the PCM cell cross section. 10 nm of PCM alloy, namely GST, is deposited on top of the silicon waveguide, followed by a 10 nm layer of indium tin oxide (ITO), which is employed as a protective film to prevent GST oxidation. **c** Example of transmission spectrum of a silicon MRR (*through* port) showing multiple quasi-periodic resonance dips, measured using grating couplers. **d** Linear response of a plastic node (RR spectra showing resonance dips at the *through* output port) for different GST crystallinity levels, obtained by insertion of a single (amorphization) or multiple (crystallization) optical pulses. **e** Resonance spectrum of a plastic node, showing the non-volatile effect of single-pulse amorphization (from “Crystalline 1” to “Amorphous”) and single-waveform recrystallization (from “Amorphous” to “Crystalline 2”).

By suitably setting the power of the input optical pulses we obtained different GST amorphization levels, which can be considered as intermediate memory states, each presenting a different resonance dip in the acquired MRR spectrum at the *through* port (Figure S1d). The chosen pulse wavelength is 0.06 nm larger than the resonance wavelength (corresponding to the center of the spectrum dip) when the GST is fully crystalline. In accordance to MRR theory, we ascribe the resonance dip changes in width and depth to variations of the GST cell absorption: a higher GST crystallization level implies a higher optical absorption, thus a larger width and a smaller depth of the resonance dip. Moreover, the GST solid-state phase also affects the effective refractive index of the corresponding waveguide segment, which in turns modifies the resonance wavelength. In particular, a larger crystalline fraction implies a larger effective refractive index and thus a larger resonance wavelength. Importantly, this effect further increases the achievable optical contrast due to different memory states, and therefore represents an additional advantage of employing a GST cell on a MRR rather than on a straight waveguide, whose transmission is much less affected by variations in effective refractive index. It should be stressed that we achieved a significantly higher optical contrast due to memory operations when experimentally investigating our plastic node compared to what we previously predicted through simulations [1] (probably because of inaccuracies in the material parameters used).

Let us now consider an example showing the maximum optical contrast, due to GST amorphization, that is achievable with a single pulse and is reversible using a single recrystallization waveform (comprising several pulses). In Figure S1e, we plotted the resonance spectra of a plastic node, corresponding to the following memory states: initial crystalline GST state (labelled as "Crystalline 1" in the legend); partially amorphized GST after the insertion of an amorphization pulse of around $14\,\mathrm{mW}$ peak power, conveying around $14\,\mathrm{nJ}$ of energy (labelled as "Amorphous" in the legend); return to initial crystalline state, after the insertion of a recrystallization waveform, consisting of pulses with around $1\,\mathrm{mW}$ peak power, conveying a total energy equal to around $1\,\mathrm{nJ}$ (labelled as "Crystalline 2" in the legend). In this case, a reversible optical contrast in the output power at 1547.4 nm greater than 40% is achieved. In comparison, to achieve a reversible optical contrast of 15% with a GST cell on a straight silicon waveguide (i.e. without the advantages of exploiting the MRR resonant behaviour), an amorphization pulse $100\,\mathrm{ns}$ long and with $1.6\,\mathrm{nJ}$ energy is required, and a recrystallization double-step pulse, $530\,\mathrm{ns}$ long and with $3.6\,\mathrm{nJ}$ energy [1]. Therefore, our plastic node shows the following improvements w.r.t. its straight waveguide counterpart: more than twice optical contrast; more than a factor 10 in amorphization energy efficiency; almost a factor 4 in recrystallization energy efficiency; a factor 10 in amorphization speed. The considered recrystallization operation, instead, is more than 3 times slower (although we did not try to maximize the speed), because it employs a sequence of single-step pulses, instead of double-step pulses. The fact that we do not need specially constructed pulses is key for the network plasticity property howere, since we want the same input shape to be able to change the memory states of the plastic nodes in both directions, allowing for a more flexible network adaptability.

2 Nonlinear response of a silicon MRR (without PCM)

In this section we demonstrate the nonlinearity and the fading memory of the photonic neuron considered in this research, namely the silicon MRR without PCM. To do so, we present an experimental measurement of the nonlinear optical transmission of a single MRR (Figure S2, a). For the theory and more in-depth analysis on the nonlinear MRR response, we refer the reader to [4, 5]. Figure S2 shows spectra of the MRR resonance measured using different input power levels. In particular, we obtained each spectra by gradually increasing the input laser wavelength and by recording the corresponding output power. It can be noticed that, for high enough input powers, the Lorentzian shape of the resonance dip is distorted, gradually approaching a triangular-like shape and with its minimum gradually redshifted as the input power increases. This well-known outcome is caused by the thermo-optic effect in silicon, where MRR temperature increases due to the MRR excitation, in turn moving the resonance wavelength towards the red. As we explained earlier, this effect is responsible for the long-term volatile memory of our artificial neurons. In the shown spectra, instead, the effect of short-term volatile memory due to free carriers is not directly visible as it is overcome by the thermo-optic effect, which is stronger at the relatively large timescale of each spectrum point acquisition.

Instead, both short- and long-term memory effects can be appreciated by looking at the nonlinear transmission of a time-dependent input waveform, such as in the example shown in Figure S2 c. Here we compare the MRR response to two different input bit sequences (blue lines, 1011101 on the left column and 1010111 on the right), for two different input power levels (namely 3.4 mW and 5.4 mW peak power, odd and even rows respectively) and for two different laser wavelengths (1549.78 nm and 1549.72 nm, first two rows and last two respectively). We notice that short-term nonlinear memory affects the transmission within the time of a bit duration ($10\,\mathrm{ns}$). For example, in the sequences on the left column, the first bit is transmitted with a decreasing trend in all but the last row, where instead the transmitted power increases again after its initial decrease. Furthermore, longer-term memory connects bits at different times, making the MRR response strongly dependent on past input bits. This effect can be easily noticed by comparing the two different sequences on the two columns in Figure S2 c. Indeed, while the response to the first high bit is the same across the two columns, we obtained different responses to the subsequent single high bit, and also to the group of three high bits, as their order is changed. As expected, these nonlinear transformations strongly depend on the input wavelength and power.

3 Further results on plastic self-adaptation

3.1 Plastic adaptation dependency on input optical power

In this section we show how self-adaptation in coupled plastic nodes (MRRs with PCM) is triggered only for a high enough input laser power. In particular, we consider a triangular PPRRNN like the one depicted in Figure 1 b in the main text, where all MRRs have a GST patch, i.e. they all are plastic nodes (Figure S3 a and b). Like in the measurements presented in Section 3 of the main text, we alternate inference and plastic adaptation (PA) steps (see Figure 2 a in the main text), employing the seventh input and the seventh output port of the triangular PPRRNN. We consider the total transmitted energy (by integrating the output waveform) in the different inference steps, grouping together in the same value distribution the different classes and 10 repetitions of the waveforms acquisition. Therefore, for each inference

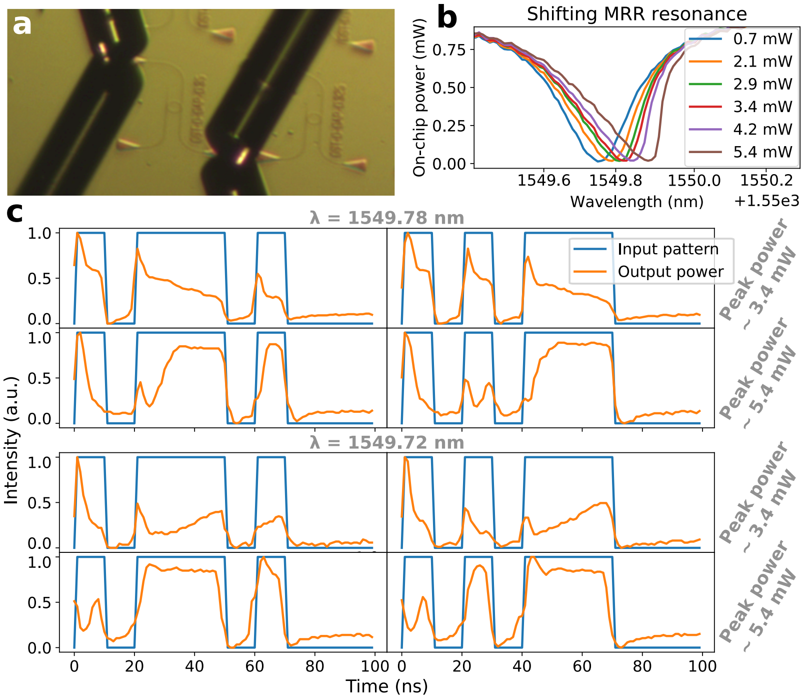

Figure S2: **Nonlinear behaviour of a single silicon MRR, without PCM.** **a** Microscope image of a single MRR during a measurement. **b** Single MRR spectra at different laser power levels, taken by progressively increasing the laser wavelength. **c** Nonlinear response (orange data) of the MRR to two different bit sequences (blue data) employed to modulate the input laser. The first two plot rows correspond to a laser wavelength of 1549.78 nm, the latter two to 1549.72 nm. Odd plot rows show the response using 3.4 mW peak laser power, even plot rows using 5.4 mW peak laser power.

step we obtain a distribution of 50 waveform energies (5 classes times 10 acquisitions). In Figure S3 c we plot the median and other quantiles (see legend) of these distributions as a function of the PA steps, for three different laser wavelengths (columns) and four different waveform average power levels (rows). Due to a technical issue, while we display the results from 6 inference steps in the first two plot rows, we can only show 5 inference steps in the last two rows. This, however, does not hinder our analysis.

It can be noticed that, for the first two input power levels (first two columns in Figure S3 c), the PA steps do not significantly modify the transmission of the bit sequences, since the observed variations of the median are comparable with the displayed quantile ranges, which represent both the variations due to acquisition noise and inter-class variations. Instead, at a higher waveform power (last two columns), the variations of the median are much larger than the displayed quantile ranges, implying that the PA steps imparted strong and non-volatile modifications to the transmission of the network. This result demonstrates that the input power threshold for the non-volatile effects (i.e. switching the GST cells) is considerably larger than then power threshold for the volatile nonlinear effects (see Figure S2 c). Thus, we confirm the scheme showed in Figure 1 a in the main text, where self-adaptation enables non-volatile tuning of volatile nonlinear responses.

3.2 Additional statistical study on self-adaptation

In this supplementary section we provide an expansion of the information given in Section 3 of the main text, regarding the plasticity introduced in the proposed photonic plastic recurrent resonator neural network (PPRRNN) by the MRRs with PCM. In particular, here we present histograms that summarize important properties of the plastic response over all the measurement sessions performed (listed in Table 2 in the main text).

In order to give an overview of how different the output feature variations are due to different input waveforms (i.e. due to plastic adapatation (PA) steps from different classes), we calculate the Pearson correlation coefficient between each pair of median output feature variation in each PA step sequence. E.g., looking at Figure 2e in the main text, the correlation is calculated between each possible pair of variations due to different PA steps in a sequence, represented in the boxes along one row. This is done considering only the measurement sessions with multiple output ports (first

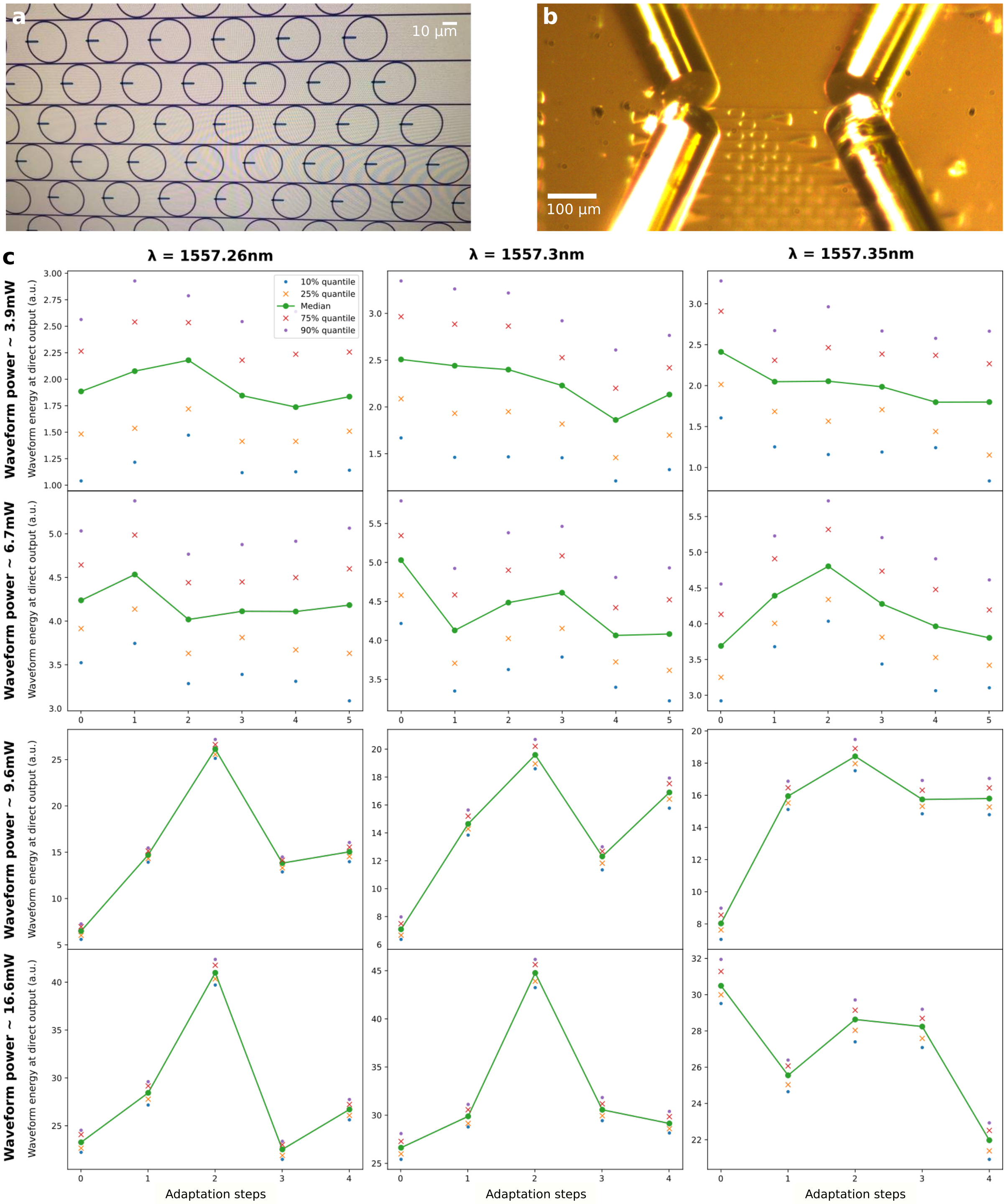

Figure S3: **Plastic adaptation at different input power levels.** **a** Micrographs of the measured triangular PPRRNN. A GST patch can be seen on every MRR. **b** Micrograph of the PPRRNN during a measurement. **c** Plots showing the median output waveform energy (together with four quantiles, see legend) of different inference steps alternated with different PA steps (x axis). Thus, significant plastic self-adaptation is detected only when the median variations across the PA steps are larger than the displayed quantile ranges. Plot rows and columns correspond, respectively, to different laser power levels and different wavelengths, as shown in bold text. It can be noticed that significant non-volatile self-adaptation happens only for the two higher laser power levels.

three in Table 2 in the main text). Then, we represent all the correlation values in a histogram (Figure S4a). We can notice that, indeed, a significant portion of the investigated PA step pairs provide output feature variations with low correlations. In particular, 596 correlation coefficients of a total of 2746 are between -0.5 and 0.5 (this correlation range is arbitrarily chosen, to provide some intuitive insight). Therefore, the PPRRNN plastic response shows *richness*, as it can vary significantly depending on the input history. Moreover, it should be noticed that a large number of pairs are highly anti-correlated, even exceeding the number of highly correlated pairs. This means that the variations due to a PA step are often reverted by another PA step in the same series. In other words, this shows that the plastic weights configuration in the PPRRNN can be often reset by a single PA step.

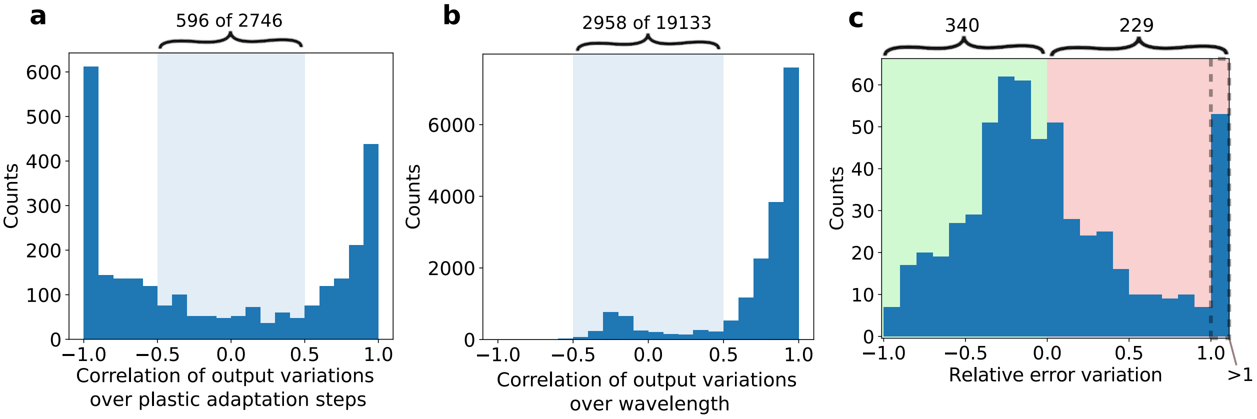

Figure S4: **Results of plasticity investigation. a, b** Histograms of the correlation coefficients between pairs of output feature variations, respectively for different PA steps and wavelengths. **c** Histogram of the error rate relative variations w.r.t. the initial error, in each sequence of PA steps.

We then performed a similar analysis by calculating the output feature variation correlation for different wavelength pairs, in the same measurement session and for a fixed PA step. E.g., looking at Figure 2e in the main text, the correlation between bar plot boxes along the same column is estimated. Also in this case, we can see that a significant portion of variation pairs present low correlation (2958 of a total of 19133 have correlation between -0.5 and 0.5). This means that PA can present very different outcomes for different wavelengths of the input optical signal. On the other hand, it is not surprising that most of the pairs are highly correlated, since the minimum wavelength difference is only $0.005\,\mathrm{nm}$ and the maximum is $0.1\,\mathrm{nm}$, to be compared with the width (at half minimum) of a MRR resonance dip, which is larger than $0.2\,\mathrm{nm}$.

We now discuss our analysis of the machine learning (ML) classification results obtained for each measurement session, each wavelength and each PA step. Mainly, we are interested in seeing how different achieved plastic weights configurations result in different ML performance, i.e. in different performance of the PPRRNN when employed to provide useful data representation to be fed to a linear classifier. In addition to the three measurement sessions considered before in this section (first three in Table 2 in the main text), our analysis now comprises also measurement sessions where a single output port and a single wavelength are used, so to allow more (30) PA steps without exceedingly increasing the measurement duration (in Table 2 in the main text, from session 4 to 12). In order to provide an overall picture of the impact of the PA step in all the considered sequences, we show a histogram (Figure S4c) of the error rate variations relative to the corresponding initial error rate value, due to each single PA step in all the measurement sessions. It can be noticed that the value distribution is centered at negative relative error variations, showing that it was more frequent that a PA step resulted in a better ML performance w.r.t. the starting value, rather than the opposite. The counts in the negative relative error variation range are 340, against the 229 in the positive range. It should be considered that, since the plotted values are relative variations w.r.t. the initial value and they cannot be less than -1 (the error rate always being a positive number), the mean of the distribution is skewed towards positive values, which explains the highly populated tail consisting of values larger than 1.

4 Neural network structure and learning process

In this section we complement the description of the neural network and of the learning procedure (chaining ensemble of RC systems) presented in the main text, namely in Section 4 and further described in Section 7.4. In particular, in Figure 4 we show an example of the first 3 chaining steps. In the first step, a readout layer (i..e a linear classifier, namely

a logistic regression) is applied and trained on each nonlinear representation. Then, the best representation-readout pair (considered as an RC system) is selected as the first element of the chain. Similarly, in the second step we select the best RC system to be added to the chain, and so on, until the desired chain length is reached.

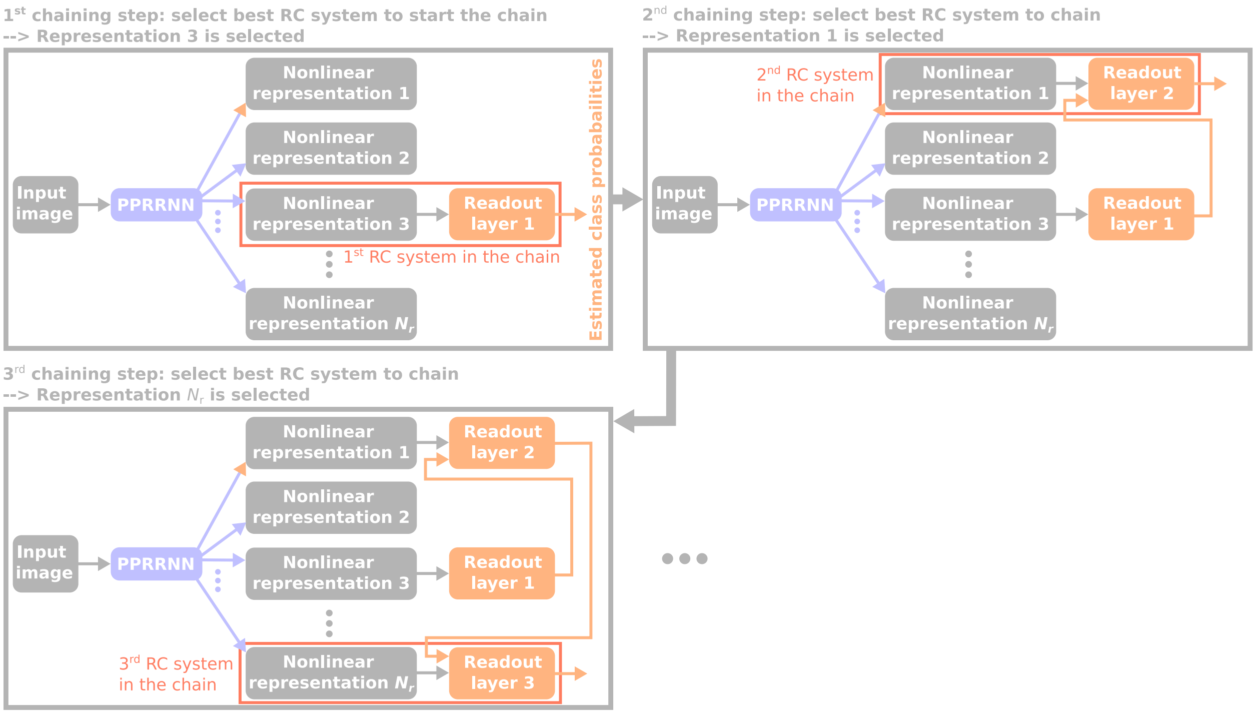

Figure S5: **Diagram of the neural network chaining procedure.** Example of 3 initial steps of the training procedure described in Section 4 and Section 7.4 in the main text. In this example we assume that, among the N_r nonlinear representations of the input data, representations number 3, 1 and N_r were respectively selected in the first 3 chaining steps. It should be noticed that, for each step, the number of training samples gradually increased at the expense of the number of validation samples, as depicted in Figure 3 b in the main text.

We now describe the neural network functioning in a more formal way, to better clarify the operations performed by the PPRRNN. Generally, we can see the input of the PPRRNN, which consists of modulated laser signals, as a collection of optical time-dependent signals, potentially one for each input port and input wavelength. Since the modulation frequencies are much lower than the light frequency, we can employ the slowly varying envelope approximation and thus we can describe each optical signal as a complex function of time, accounting for both optical amplitude and phase. Therefore, we write the PPRRNN input $x(p_{in}, t, \lambda)$ as a complex function of the input port number $p_{in} = 1, 2, 3, ...$, of time *t* and of the input wavelength λ. Note that in the experiments presented in this work, we always insert only a time series at a time, each into a fixed input port and with fixed wavelength, and we directly modulate only the amplitude of the input laser light. Moreover, we can describe the operation of each node in the PPRRNN as:

$$y_o(t, \lambda) = S_{1,o}(t, \lambda, \Phi)x_1(t, \lambda) + S_{2,o}(t, \lambda, \Phi)x_2(t, \lambda)$$

where y_o denotes the complex node output at its output port o, x_1 and x_2 the complex inputs at node output port 1 and 2, respectively, $S_{1,o}$ and $S_{2,o}$ the complex scatter matrix elements connecting the input port 1 and 2, respectively, to the output port o, and Φ fixed parameters linked to the MRR design and fabrication, such as the cold resonance wavelength. In other words, at a given time and for a fixed input wavelength, each node simply combines its inputs after multiplying them by a complex number, which modulates optical amplitude and phase. Since each node is affected by optical losses and there is no amplification, we have that $|S| < 1$.

The actual complexity of the node response lies in its time dependency, which is mainly the result of the MRR resonance perturbations due to ring temperature, free-carrier density, PCM solid state phase and temperature (if the node has a PCM cell), as explained in Sections 1 and 2. These quantities connect optical inputs at different times (memory) and at different wavelengths, and are best modelled by differential equations. For the details of such models and simulations we refer the reader to [1, 4, 2]. Finally, it should be stressed that the loops in the PPRRNN topology do not introduce memory in practice, as the time of light travel through the system is negligible w.r.t. the input signal timescale. Therefore, optical signals can be considered to propagate instantaneously through the PPRRNN.

References

[1] Alessio Lugnan, Santiago García-Cuevas Carrillo, C David Wright, and Peter Bienstman. Rigorous dynamic model of a silicon ring resonator with phase change material for a neuromorphic node. *Optics Express*, 30(14):25177–25194, 2022.

[2] Santiago García-Cuevas Carrillo, Alessio Lugnan, Emanuele Gemo, Peter Bienstman, Wolfram HP Pernice, Harish Bhaskaran, and C David Wright. System-level simulation for integrated phase-change photonics. *Journal of Lightwave Technology*, 39(20):6392–6402, 2021.

[3] Xuan Li, Nathan Youngblood, Zengguang Cheng, Santiago Garcia-Cuevas Carrillo, Emanuele Gemo, Wolfram HP Pernice, C David Wright, and Harish Bhaskaran. Experimental investigation of silicon and silicon nitride platforms for phase-change photonic in-memory computing. *Optica*, 7(3):218–225, 2020.

[4] Thomas Van Vaerenbergh, Martin Fiers, Pauline Mechet, Thijs Spuesens, Rajesh Kumar, Geert Morthier, Benjamin Schrauwen, Joni Dambre, and Peter Bienstman. Cascadable excitability in microrings. *Optics express*, 20(18):20292–20308, 2012.

[5] Stefano Biasi, Giovanni Donati, Alessio Lugnan, Mattia Mancinelli, Emiliano Staffoli, and Lorenzo Pavesi. Photonic neural networks based on integrated silicon microresonators. *arXiv preprint arXiv:2306.04779*, 2023.
